# Genomic regions and candidate genes selected during the breeding of rice in Vietnam

**DOI:** 10.1111/eva.13433

**Published:** 2022-07-09

**Authors:** Janet Higgins, Bruno Santos, Tran Dang Khanh, Khuat Huu Trung, Tran Duy Duong, Nguyen Thi Phuong Doai, Anthony Hall, Sarah Dyer, Le Huy Ham, Mario Caccamo, Jose De Vega

**Affiliations:** ^1^ Earlham Institute Norwich UK; ^2^ NIAB Cambridge UK; ^3^ Agriculture Genetics Institute (AGI) Hanoi Vietnam; ^4^ Vietnam National University of Agriculture Hanoi Vietnam

**Keywords:** allele mining, genome scan, landraces, rice, selection

## Abstract

Vietnam harnesses a rich diversity of rice landraces adapted to a range of conditions, which constitute a largely untapped source of diversity for the continuous improvement of cultivars. We previously identified a strong population structure in Vietnamese rice, which is captured in five Indica and four Japonica subpopulations, including an outlying *Indica‐5* group. Here, we leveraged that strong differentiation and 672 native rice genomes to identify genomic regions and genes putatively selected during the breeding of rice in Vietnam. We identified significant distorted patterns in allele frequency (XP‐CLR) and population differentiation scores (*F*_ST_) resulting from differential selective pressures between native subpopulations, and later annotated them with QTLs previously identified by GWAS in the same panel. We particularly focussed on the outlying *Indica‐5* subpopulation because of its likely novelty and differential evolution, where we annotated 52 selected regions, which represented 8.1% of the rice genome. We annotated the 4576 genes in these regions and selected 65 candidate genes as promising breeding targets, several of which harboured alleles with nonsynonymous substitutions. Our results highlight genomic differences between traditional Vietnamese landraces, which are likely the product of adaption to multiple environmental conditions and regional culinary preferences in a very diverse country. We also verified the applicability of this genome scanning approach to identify potential regions harbouring novel loci and alleles to breed a new generation of sustainable and resilient rice.

## 1 INTRODUCTION

Vietnam harnesses a rich novel rice diversity due to the presence of native and traditional rice varieties adapted to its broad latitudinal range, diversity of ecosystems and regional food preferences (Fukuoka et al., 2003). This diversity constitutes a largely untapped and highly valuable genetic resource for local and international breeding programs (Khanh et al., 2021). Vietnamese rice shows a strong population structure, which is captured within five Indica and four Japonica subpopulations that we have recently described (Tables 1 and 2; Higgins et al., 2021). These subpopulations were characterized in relation to the fifteen subpopulations of Asian rice described by the rice 3000 rice genomes project (3K RGP; Zhou et al., 2020). Among these nine populations described in Vietnam, the *Indica‐5* (I5) subpopulation is an outlier and is expanded in Vietnam and, therefore, a potential source of novel variation compared with the wider Asian diversity.

**TABLE 1:** Number of accessions in each subpopulation by region of collection and basic description of each subpopulation

Subtype	Indica						Japonica				
Subpop.	I1	I2	I3	I4	I5	Im	J1	J2	J3	J4	Jm
Total	145	91	37	62	43	48	115	50	17	21	8
**π**	0.0144	0.00127	0.0012	0.0012	0.001	‐	0.0006	0.0005	0.0007	0.0005	‐
Region of collection (administrative regions of Vietnam)											
Northeast	5	1	7	1	2	5	22	13	0	1	1
Northwest	4	1	14	5	0	7	55	11	1	0	0
Red River Delta	6	1	0	32	12	5	0	6	0	8	0
North Central Coast	5	0	6	9	13	2	34	4	1	3	2
South Central Coast	3	1	8	2	4	13	0	1	12	0	0
Central Highlands	1	0	0	0	0	0	0	0	0	0	2
Southeast	1	3	1	0	0	0	0	1	1	0	0
Mekong Delta	15	44	0	0	0	0	0	0	0	0	0
Unknown	105	40	1	13	12	12	4	14	2	9	3
Dataset											
New^a^	135	77	36	52	38	41	113	47	16	20	6
3KRGB^b^	10	14	1	10	5	7	2	3	1	1	2

^a^ *New*: Accession newly sequenced by us in Higgins et al. (2021). *3KRGP*: Accessions sequenced in Zhou et al. (2020) by the *3000 Rice Genome Project*. (**π**) Mean nucleotide diversity of each subpopulation. Regions sorted from North to South.

^b^ Descriptors from Higgins et al. (2021): Short‐growth: growth‐duration (less than 120 days from sowing to harvest). Long‐growth: long growth‐duration (over 140 days for sowing to harvest).

**TABLE 2:** Subpopulation descriptions summary, based on Higgins et al. (2021)

Subtype	Subpopulation	Agromorphology	3K‐RGP overlap^a^
Indica	I1	Elite cultivars, Short season (<120 days), irrigated, lowland, longer grains, earlier heading date, higher culm strength, shorter leaf length, shorter culm length	XI‐1B1 (modern varieties), a few admixed (XI‐adm)
	I2	Landraces, Long season (<120 days), tall, rainfed, Mekong Delta	XI‐3B1
	I3	Landraces, Upland, deep roots	XI‐3B1, XI‐3B2
	I4	Landraces, Rainfed lowland, Red River Delta	XI‐3B2
	I5	Landraces, Northern and Red River Delta, lowland, thin roots, low genetic diversity, small non‐glutinous grains	XI‐adm
Japonica	J1	Tropical, Upland, North Vietnam, rainfed	GJ‐sbtrp
	J2	Temperate, Lowland, short grains, broad range, irrigated, lower grain/width length	GJ‐tmp
	J3	Subtropical, Upland, large grains, South Central Coast	GJ‐sbtrp, GJ‐trp1, GJ‐adm
	J4	Temperate, Lowland, short grains, Red River Delta, irrigated	GJ‐tmp

^a^ Classification of accessions shared between Higgins et al. (2021) and the 3000 Rice Genome Project, which allowed to compare both population structures.

Genetic variation and differentiation are influenced by natural processes, such as adaption and random drift, as well as conscious systematic breeding selection and unconscious selection by producers, due to the agricultural practices of local farmers. Selection causes detectable changes in allele frequencies at the selected sites and their flanking regions. By modelling differences in allele frequency in close loci between neutrality and selection scenarios, the cross‐population composite likelihood ratio test (XP‐CLR) can detect selective sweeps (Chen et al., 2010), making it one of the popular options to detect natural selection in genomic data (Vitti et al., 2013). Any distorted pattern in allele frequency in contiguous SNP sites would have occurred too quickly (speed of change is assessed over expanding windows based on the length of the affected region) to be explained by random drift (Chen et al., 2010). XP‐CLR can detect both hard sweeps, where a single beneficial mutation at a given locus rapidly increases in frequency as a result of selection, and soft sweeps, which are present in multiple genetic backgrounds before being subject to selection, making them harder to detect (Hartfield et al., 2017; Hartfield & Bataillon, 2020; Lai et al., 2018). Therefore, XP‐CLR is a powerful approach to identify the putative signals underlying local adaption and delineate candidate regions, and part of identification pipelines that include later data integration with QTLs, *F*_ST_ and nucleotide diversity scores. This approach has been used to identify regions of selection associated with domestication and improvement in a wide range of both autogamous and outcrossing crops, for example apple (Duan et al., 2017), soybean (Zhou et al., 2015), maize and sorghum (Lai et al., 2018), cucumber (Qi et al., 2013), spinach (Gyawali et al., 2021) and wheat (Joukhadar et al., 2019). The qualitative patterns of different selective sweeps showed similar in outcrossed and autogamous species, yet stretched over larger chromosomal regions in the latter (Hartfield & Bataillon, 2020).

XP‐CLR has proved a popular method in rice to detect both past and recent selection signatures of domestication. Lyu et al. (2014) identified a list of differentiated genes that may account for the phenotypic and physiological differences between upland and irrigated rice. Xie et al. (2015) compared Indica semi‐dwarf modern‐bred varieties (IndII) with taller Chinese landraces (IndI) to identify signatures of rice improvement and detected 200 regions spanning 7.8% of the genome. Meyer et al. (2016) identified genomic regions associated with adaptive differentiation between *O. glaberrima* populations in Africa. He et al. (2017) tested for positive selection between weedy and landrace rice using five different approaches. Cui et al. (2020) identified potential selective sweeps in both Indica and Japonica genomes showing that there were multiple loci responding to selection and that loci associated with agronomic traits were particularly targeted by selection. Lyu et al. (2014) used XP‐CLR to demonstrate how introgressed regions were selected through hybrid rice breeding. Xiao et al. (2021) determined whether GWAS‐mapped genes were artificially selected during the breeding process in Japonica rice. While these studies were trying to answer different questions, all used XP‐CLR to detect selected regions. In addition, many of the studies used other metrics, such as the fixation index (*F*_ST_), to verify selected regions.

Here, we identified regions in the rice genome which have been selected by conscious and unconscious human selection by leveraging the strong population structure among Vietnamese‐native rice varieties and landraces, which has resulted from adaptation to diverse geography, environmental pressures and agronomic practices. Rice has been cultivated in Vietnam for over 4000 years (Khanh et al., 2021) and originated around 9000 years ago from the Yangtze valley (Gutaker et al., 2020). Selection within Vietnam has resulted in the four Japonica and five Indica subpopulations, these are comprised of landraces except for the I1 subpopulation, which is comprised of accessions with ‘elite’ genetic composition, resulting from recent breeding with modern‐bred varieties (Tables 1 and 2; Higgins et al., 2021).

Unravelling the genomic differences and identifying regions selected between these nine subpopulations is the first step towards understanding their breeding potential. We focussed on the outlying *indica‐5* (I5) subpopulation to identify candidate loci for breeding targets, as this subpopulation constitutes a gene‐pool not used in rice improvement. To assess the putative role of these selected regions and whether these selected regions may contain loci that potentially could control agronomic traits, we looked for overlaps with previously mapped QTLs in the same diversity panel, and regions enriched in gene ontology (GO) terms. QTLs have been described for a range of agronomic traits using the complete set of 672 native rice accessions (Higgins et al., 2021), while a subset of 182 of these traditional Vietnamese accessions (Phung et al., 2014) was used for genome‐wide phenotype–genotype association studies (GWAS) relating to root development (Phung et al., 2016), panicle architecture (Ta et al., 2018), drought tolerance (Hoang, van Dinh, et al., 2019), leaf development (Hoang et al., 2019), Jasmonate regulation (To et al., 2019) and phosphate starvation and efficiency (Mai et al., 2020; To et al., 2020). Finally, we studied alleles with nonsynonymous substitutions in candidate genes in selected regions of the outlying and highly selected I5 subpopulation.

## 2 MATERIALS AND METHODS

### 2.1 Sequencing and SNP calling and annotation

We sequenced 616 Vietnamese samples and incorporated 56 samples from the ‘3000 Rice Genomes Project’ (3K RGP) that originated from Vietnam, to give a total of 672 samples. Plant accessions were obtained from the Vietnamese National Genebank in compliance with the national laws and international treaties. The 616 rice samples were mapped to the Japonica Nipponbare (IRGSP‐1.0) reference with BWA‐MEM using default parameters, duplicate reads were removed with Picard tools (v1.128) and the Bam files were merged using SAMtools v1.5. Variant calling was completed on the merged Bam file with FreeBayes v1.0.2 using the option ‘‐‐min‐coverage 10’. Over 6.3 M bi‐allelic SNPs with a minimum allele count of three and quality value above 30 and missing genotype calls in under 50% of samples were obtained with VCFtools v0.1.13. Read alignments to the Nipponbare IRGSP 1.0 reference genome in Bam format were downloaded from http://snp‐seek.irri.org/ (Mansueto et al., 2017) for the samples from the 3K RGP. These Bam files were directly merged, as variant calling had been similarly completed using FreeBayes v1.0.2 (Garrison & Marth, 2012), for each of the 12 chromosomes using the option ‐‐min‐coverage 10, and filtered with VCFtools v0.1.13 as before, to obtain 6.8 M bi‐allelic SNPs. The two sets of 6.3 and 6.8 M SNPs were merged using BCFtools isec v1.3.1 to obtain 4.4 M SNPs which were present in both sets and in at least 70% of samples. These 4.4 M SNPs were then filtered to remove positions which fell outside the expected level of heterozygosity for this data set, using a cut‐off value of 0.591 (Higgins et al., 2021), which resulted in 3.8 M SNPs passing this filter. Missing data were imputed in this latest dataset using Beagle v4.1 with default parameters (Browning & Browning, 2016). Two separate SNP sets were generated, one for the 426 Indica sample and another for the 211 Japonica samples, each of these SNP sets was subsequently filtered for a minor allele frequency of 5%, to give a set of 2,027,294 SNPs for the 426 Indica samples and 1,125,716 SNPs for the 211 Japonica samples. Passport information for each sample is available in Higgins et al. (2021). A summary of the number and source of each subpopulation is available in Table 1 (47 Indica samples and 9 Japonica samples native to Vietnam were obtained from the 3K RGP project) and the proportion of the samples collected from each of the eight regions in Vietnam is plotted in the Appendix S1. The putative functional effects of the bi‐allelic SNPs (low, medium and high effects) on the genome were determined using SnpEff (Cingolani et al., 2012) and the prebuilt release 7.0 annotation from the Rice Genome Annotation Project (http://rice.plantbiology.msu.edu/) as detailed in (Higgins et al., 2021).

### 2.2 Identification of selective sweeps using XP‐CLR

Selective sweeps across the genome were identified using XP‐CLR (Chen et al., 2010), a method based on modelling the likelihood of multilocus allele frequency differentiation between two populations. An updated version of the original code was used (https://github.com/hardingnj/xpclr). We used 100 kbps sliding windows with a step size of 10 kbps and the default option of a maximum of 200 SNPs in any window. XP‐CLR was run comparing the five Indica subpopulations to each other and the four Japonica subpopulations to each other. Selected regions were extracted using the XP‐CLR score for each 100 kbps window as follows: 200 kbps centromeric regions were removed. The mean and 99th percentile of the XP‐CLR scores were calculated for each comparison between one subpopulation against the remaining ones (e.g. I5 vs. I1, I2, I3 and I4). The mean 99th percentile was used to define the cut‐off level for selection in that subpopulation. 100 kbps regions with an XP‐CLR score higher than the cut‐off were extracted and contiguous regions were merged using BEDTools v2.26.0 (Quinlan & Hall, 2010) specifying a maximum distance between regions of 100 kbps. Regions shorter than 80 kbps were removed to give a final set of putatively selected regions for each comparison. Putative regions observed selected in at least two comparisons for Japonica subpopulations, or three comparisons for Indica subpopulations, were merged to obtain a final set of selected regions for each subpopulation. BEDTools map was used for finding any overlap of selected regions with QTLs. QTL regions using the same, or a subset of, the samples were previously identified by reviewing the literature. Genes lying within the selected regions were extracted and checked for enrichment in Protein Domain and Pathway using a maximum Bonferroni FDR value of 0.05 in PhytoMine (https://phytozome.jgi.doe.gov/), a service implemented within Phytozome (Goodstein et al., 2012).

### 2.3 Calculating *F*_ST_

We calculated *F*_ST_ per SNP between the 43 samples in the I5 subpopulation and the 190 samples in the I2, I3 and I4 subpopulations with VCFtools using the ‘weir‐fst‐pop’ option, which calculates *F*_ST_ according to the method of Weir and Cockerham (Weir & Cockerham, 1984). *F*_ST_ was calculated both for individual SNPs and over 100,000 bp sliding windows with a step size of 10,000 bp. Sites which are homozygous between these populations were removed, and negative values were changed to zero. The mean *F*_ST_ was calculated per gene and per specified region.

### 2.4 Enrichment analysis of GO terms in selected regions

The enrichment analysis was made with the library topGO (Alexa, 2010) in R, using as inputs the lists of genes in each selected region, and the functional annotation of the rice genome (Rice MSU7.0) from agriGO (http://bioinfo.cau.edu.cn/agriGO). The method in topGO compared the genes observed in each selected region annotated with a given GO term with the expected number of genes annotated with that term in the whole transcriptome. The statistical test was a F‐Fisher test (FDR <0.05) with the ‘weight01’ algorithm in topGO. The ‘weight01’ algorithm resolves the relations between related GO ontology terms at different levels. The selected regions with over‐represented GO terms, and the number of genes they contained, were plotted using ggplot2 (Wickham, 2016).

## 3 RESULTS

### 3.1 Identification of selective sweeps among Vietnamese subpopulations

To identify genomic regions that have been selected during the breeding of rice in Vietnam, we searched for genomic regions with distorted patterns of allele frequency that cannot be explained by random drift using XP‐CLR (Chen et al., 2010). We used our previously described data set of 672 genomes from Vietnamese‐native landraces and varieties, which have been divided into nine subpopulations (Tables 1 and 2; Higgins et al., 2021). We compared all the five Indica subpopulations to each other and all the four Japonica subpopulations to each other. First, we obtained the mean XP‐CLR score over the whole genome, as summarized in Table 3, with the reciprocal differences in the comparisons between each pair of subpopulations in Table S1. Among the Japonica subpopulations, the J4 subpopulation had the highest selection scores consistently, especially against the J1 subpopulation. Among the Indica subpopulations, the I1 subpopulation had the lowest selection scores consistently. The I5 subpopulation had the highest selection scores except in comparison with the I3 subpopulation. We calculated the 99th percentile for each comparison between a pair of subpopulations and used the mean value for each subpopulation as a cut‐off to identify selected regions (detailed in Table S2 and summarized in Table 4). We merged selected regions within 100 kb of each other, so the final set of selected regions for each comparison were of variable length. Selected regions were usually longer, the higher was the XP‐CLR score. The regions selected in the comparisons between a pair of subpopulations were plotted along each chromosome for the Indica subpopulations (Figure S1) and the Japonica subpopulations (Figure S2).

**TABLE 3:** Whole‐genome XP‐CLR selection scores

	SCORE	J1	J2	J3	J4
Selected	J1		17.8	7.6	6.1
	J2	19.5		21.6	6.6
	J3	24.4	17.9		5.9
	J4	46.1	17.5	17.9	

	SCORE	I1	I2	I3	I4	I5
Selected	I1		8.5	4.0	8.2	9.8
	I2	28.8		7.0	15.7	17.3
	I3	40.2	24.5		23.2	23.7
	I4	34.1	21.5	7.4		18.6
	I5	63.6	44.6	18.0	39.2	

*Note*: Mean XP‐CLR score across the whole genome for each comparison between the four Japonica subpopulations and the five Indica subpopulations. Reciprocal comparisons shown in Table S1.

**TABLE 4:** XP‐CLR scores and summary on the regions under selection in each subpopulation

	Mean XP‐CLR score	Cut‐off^a^	Regions over 80 kbp	Mean length	Total length	% genome^b^	Genes
J1	10.5	136	28	576,707	16,147,785	4.3	2427
J2	25.9	256	23	726,689	16,713,841	4.5	2439
J3	16.1	228	24	577,089	13,850,139	3.7	2007
J4	27.1	297	25	731,341	18,283,522	4.9	2643
I1	7.6	161	44	453,570	19,957,065	5.3	3077
I2	17.2	275	41	550,836	22,584,270	6.1	3346
I3	27.9	401	42	474,009	19,908,387	5.3	2993
I4	20.4	306	38	619,404	23,537,343	6.3	3465
I5	41.4	440	52	583,706	30,352,734	8.1	4576

*Note*: Individual comparisons are shown in Table S2.

^a^ Cut‐off: 99 percentile.

^b^ Rice reference genome of 373,245,519 bp.

**FIGURE 1:**
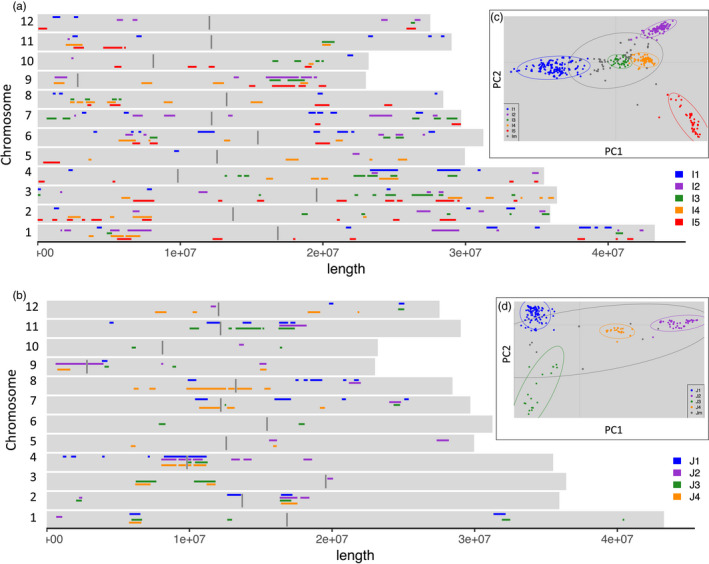
XP‐CLR scores and regions under selection. (a) Selected regions for the five Indica subpopulations covering 5.4%, 6.1%, 5.3%, 6.3% and 8.1% of the genome for I1, I2, I3, I4 and I5 respectively. Centromeric regions are shown as 100 kb regions in dark grey. (b) Selected region for the four Japonica subpopulations covering 4.3%, 4.5%, 3.7% and 4.9% of the genome for J1, J2, J3 and J4 respectively. (c) PCA showing the relationship of the five Indica subpopulations, taken from Figure 2. Higgins et al. (2021). (d) PCA showing the relationship of the four Japonica subpopulations, taken from Figure 2. Higgins et al. (2021)

To define a final set of selected regions in a given subpopulation, we retained and merged regions selected in at least three comparisons between that subpopulation and any other subpopulation in the case of the Indica ones, or in at least two comparisons in the case of the Japonica subpopulations. This procedure is described in detail for the I5 subpopulation in a subsequent section. The final set of selected regions in each subpopulation were plotted along each of the rice chromosomes in Figure 1a,b for the Indica and Japonica subtypes, respectively. The selected regions ranged from 98,583 to 2,787,579 bases for the Japonica subpopulations, and from 106,844 to 2,309,615 bases for the Indica subpopulations. We observed slightly different patterns in length variation per subtype and subpopulation (Figure S3). Overall, the Japonica subpopulations had fewer selected regions, which represented from 3.7% to 4.9% of the genome, while Indica subpopulations ranged from 5.3% to 8.1% of the genome. Gene lists for the selected regions are available in Table S3. The Japonica subtypes had a higher proportion of long selected regions. These regions were confined to specific areas of the genome and absent from large chromosome regions. All four Japonica subpopulations were selected on the long arm of chromosome 2 and in both flanks of the centromeric region of chromosome 4. The selected regions in the Indica subpopulations were spread throughout the genome and very variable in length. We particularly observed a high proportion of shorter than average selected regions and a lower proportion of longer than average selected regions in the I1 subpopulation. The I5 subpopulation stands out as having the highest proportion of the genome under selection, overlapping with the other landrace subpopulations (I2, I3 and I4) on the short arm of chromosome 1 and the long arm of chromosome 9. However, selected regions in I5 were absent on the long arm of chromosome 4, where all other landrace subpopulations overlapped with the elite I1 subpopulation.

### 3.2 Putative roles of the regions under selection

We looked for the overlap of the selected regions with sets of QTLs previously reported in the literature (Table 5; Tables S4 and S5); 21 QTLs for basic plant and seed architecture traits were identified using the same complete set of Vietnamese rice samples (Higgins et al., 2021); and 88 QTLs associated with root development traits (Phung et al., 2016), 29 QTLs for panicle morphological traits (Ta et al., 2018), 17 QTLs for tolerance to water deficit (Hoang, van Dinh, et al., 2019), 13 QTLs for leaf mass traits (Hoang, Gantet, et al., 2019), 25 QTLs for growth mediated by jasmonate (To et al., 2019), 21 QTLs for phosphate starvation (Mai et al., 2020) and 18 QTLs for phosphate efficiency (To et al., 2020) reported for a subset of 180 samples of the whole dataset.

**TABLE 5:** Putative traits selected in each subpopulation based on the overlaps between QTLs and regions, which are further detailed in Tables S4 and S5

TRAIT		INDICA					JAPONICA			
Trait ID	Description	I1	I2	I3	I4	I5^a^	J1	J2	J3	J4
GL	Grain length	6,6	6	2	6	6	2	2,4	2	2,7
GS	Grain size							3		
HD	Heading Date		9		4					
FP	Floret Pubescence				9		8			
PBintL	Primary branch internode length	7	1		1		8			
PBL	Primary branch length			8	8	8				
PBN	Primary branch number			8,10	8	8		1		
SBintL	Secondary branch internode length		12							
SBN	Secondary branch number						2	2	2	2
TIL	Number of tillers	1,7		3			7,11	11	11	
PL	Panicle length					5,6				
RL	Rachis length	4		11	4,11			9		9
SHL	Shoot length	1,12	1	1,8,11	8,11	8				
SHW	Shoot weight	1,12	12							
SpN	Spikelet number		1		1	1	2	2	2	1,2
TTW	Total weight	1	1,9,12		3,9					
RCGR	Relative crop growth rate								6	
R‐S	Root to shoot ratio			6						
DEPTH	Deepest point reached by roots	1	1		8	7		11		8,11
DRP	Deep root proportion (<40 cm)	6	1,1		1	1	1		1	1
DRW	Deep root mass (<40 cm) weight	6			1	1	1			1
DW2040	Root mass 20–40 cm	6								
DW4060	Root mass 40–60 cm	6,12								
DWB60	root mass below 60 cm				1	1	1			1
MRL	Maximum root length				5	6				
NCR	Number of crown roots	12	1	3		6,8,11	11	11	11	
RDW	Root dry weight	6								
RTL	Root length			2		2				
RTW	Root weight						11	11	10,11	
SRP	Shallow root proportion (0–20 cm)	6		4				4		
THK	Root thickness		2	2	3		11	11	11	
FW	Leaf fresh weight	1	12,12	1,10		1			6	
LLGHT	Longest leaf length			6	6	6			6	
TW	Leaf turgid weight	1	12,12	1,10		1			6	
RWC_1W	RWC after 1w drought	11								
RWC_2W	RWC after 2w drought	7,11			11	11				
RWC_3W	RWC after 3w drought	7,11			8,11	7,11				
RECO_1W	Recovery ability after 1w drought	7,11					7			
RECO_3W	Recovery ability after 3w drought	11			11	11				
RECO_4W	Recovery ability after 4w drought			11	11			5		5
RPPUE	Relative physiological phosphate use efficiency				5					
RPUpE	Relative phosphate uptake efficienc	1			3	1				

*Note*: Numbers indicate the chromosomes where the selected region(s) associated with the trait are selected.

^a^ Genes within selected regions in indica‐5 further detailed in Tables S7 to S13. RWC: relative water content. Traits description extracted from the overlapping QTL descriptions. Overlaps showed in Figure 4. QTLs from eight published studies (Higgins et al., 2021; Hoang, Gantet, et al., 2019; Hoang, van Dinh, et al., 2019; Mai et al., 2020; Phung et al., 2016; Ta et al., 2018, 2019; To et al., 2020).

The selected regions in the Japonica subpopulations had overlaps with all the QTLs sets, except QTLs associated with growth regulation by jasmonate (Tables 5 and S5). The region on chromosome 2 that was selected in all Japonica subpopulations overlapped with a QTL for grain length (2_GL) and two related QTLs for panicle morphology, secondary branch number (SBN) and spikelet number (SpN). These QTLs collocate with osa‐MIR437 (Ta et al., 2018), a monocot preferential miRNA that targets LOC_Os02g18080 (https://rapdb.dna.affrc.go.jp). J2 and J4 lowland varieties were both selected on the long arm of chromosome 5 and at the start of chromosome 9. The region on chromosome 5 overlaps with a QTL for drought sensitivity observed after 4 weeks of drought stress (q4_Score4). The selected region on chromosome 9 overlaps with a QTL for rachis length (RL), which is associated with the size of the panicle, a key component of yield. The region towards the end of chromosome 11, which was selected in J1, J2 and J3, overlaps with qRTW11.19 as well as several QTLs associated with root traits: Rq13_J_TIL, Rq29_J_DEPTH, Rq30_J_DEPTH, Rq46_F_NCR, Rq63_J_THK.

The selected regions in the Indica subtypes overlapped with all the QTL sets (Table S4). Most overlaps that occurred in more than one subpopulation were also observed in the I5 subpopulation, so are discussed in the next section. In addition, the region on the long arm of chromosome 11, which is selected in both I3 and I4, overlaps with QTLs for drought sensitivity (Tq17 Score4), rachis length (QTL25 RL) and response to jasmonate (qSHL5).

The total number of genes within the selected regions are shown in Table 4. For the Japonica subtypes, the number of genes ranged from 2007 genes within the selected regions of the J3 subpopulation to 2643 genes within the selected regions of the J4 subpopulation. For the Indica subtypes, the number of genes ranged from 2993 to 3465 in the I1 to I4 subpopulations, whilst the I5 subpopulation had 4576 genes within 52 selected regions (gene listed in Table S3). The overlap between genes selected in each subpopulation showed that around half of the genes selected in a subpopulation were unique to that subpopulation (Figure S4). No common genes were selected in all subpopulations, but 230 genes were selected in all four Japonica subpopulations, and 44 genes were selected in all the Indica landrace subpopulations I2 to I5.

**FIGURE 2:**
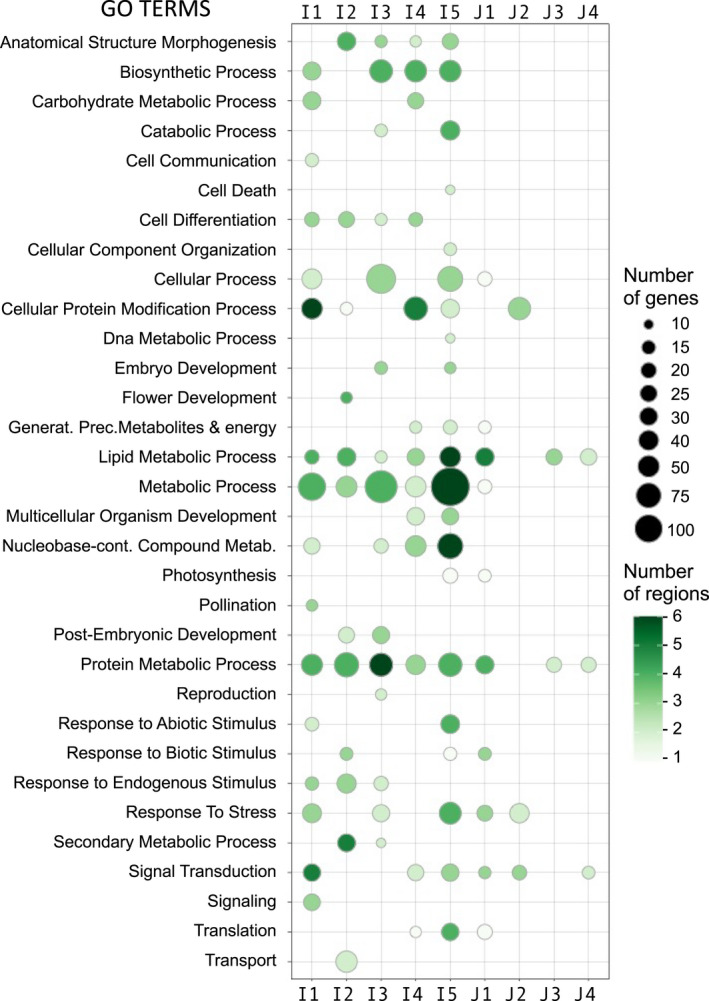
Gene Ontology overrepresentation

The enrichment analysis of the GO terms enriched in each selected region was obtained by comparing the annotations in each selected region with the whole‐genome annotation, as background (Table S6). The number of genes associated with enriched terms in different regions from the same subpopulation were added up and plotted (Figure 2). A large proportion of genes in selected regions were associated with the same biological functions in the different Indica subpopulations, for example, lipid and protein metabolic process, or ‘Biosynthetic process’. However, we also evidenced specific selections in particular subpopulations, such as ‘Photosynthesis’ genes in I5 and J1; biotic response genes in I2, I5 and J1; abiotic response genes in I1 and I5; and ‘flower development’ genes in I2. Selected regions were more clearly associated with specific GO terms in the Indica subpopulations than in the Japonica ones. The enrichment of GO terms was not correlated with the total number of genes or genome length in each subpopulation (Table S2).

### 3.3 Selected regions in the outlying Indica‐5 (I5) subpopulation

**FIGURE 3:**
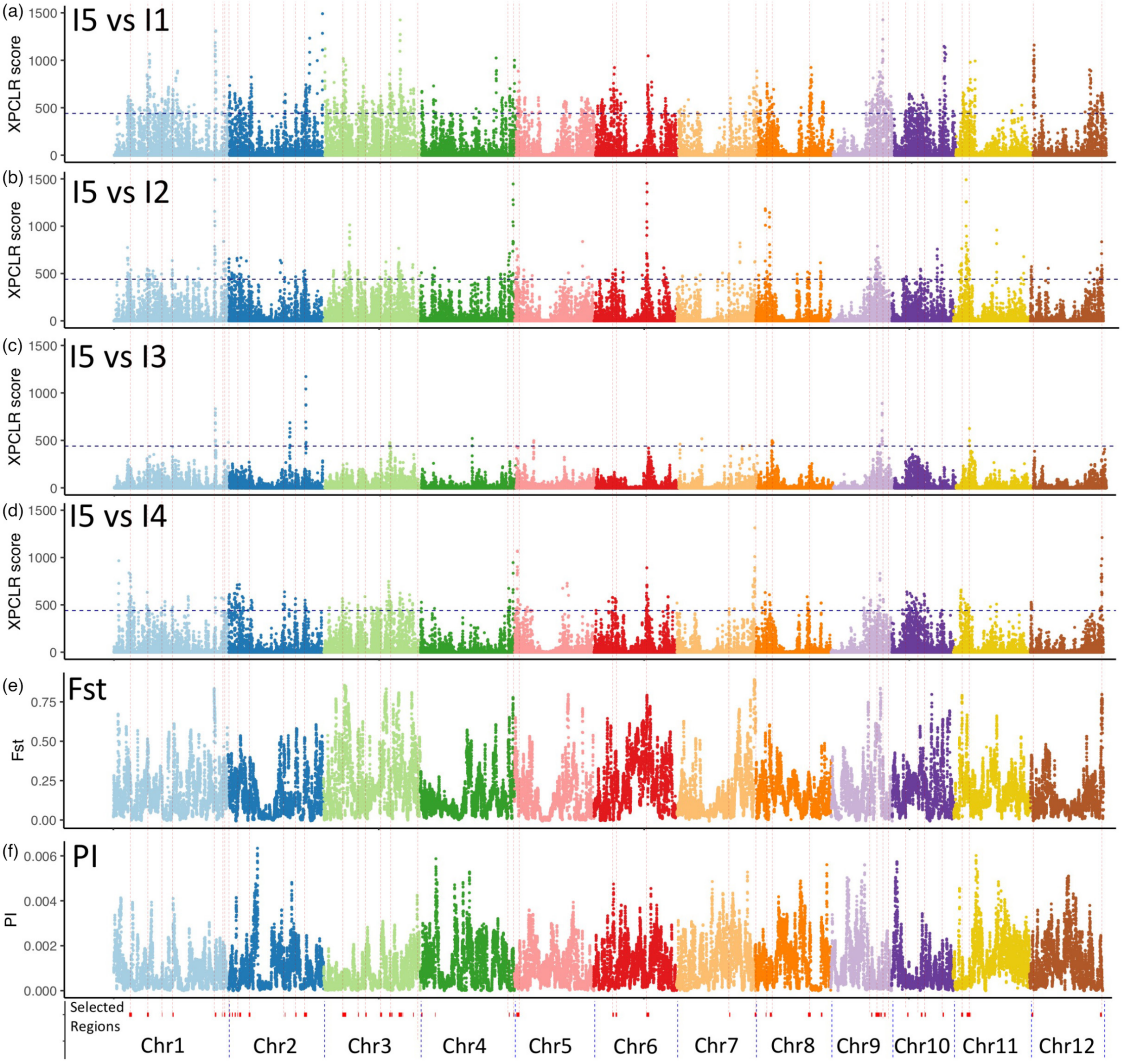
Selection sweeps in the Indica I5 subpopulation compared to the other Vietnamese subpopulations. XP‐CLR scores in 100,000 bp sliding windows are plotted along the 12 chromosomes, showing selection in the I5 subpopulation compared to (a) I2, (b) I2, (c) I3, (d) I4. The horizontal dashed line indicates the threshold XP‐CLR score of 440 for determining selected regions. (e) *F*_ST_ in 100,000 bp sliding windows for the 43 samples in the I5 subpopulation compared to the 190 samples in the I2, I3 and I4 subpopulations. The *F*_ST_ peaks (selection signatures) ranged from 0.5 and 0.8, while the average *F*_ST_ (associated with subpopulation differentiation) was 0.18 for this comparison. (f) Whole‐genome genetic diversity (Π) in 100,000 bp sliding windows for the 43 samples in the I5 subpopulation. The vertical lines show the position of the 52 selected regions

The XP‐CLR score of the I5 subpopulation compared to the other four Indica subpopulations in 100 kbps windows is shown in Figure 3. Overall, the I5 subpopulation had the highest XP‐CLR selection scores, this is reflected in I5 having the greatest number of selected regions covering the highest proportion of the genome. I5 is an outlier subpopulation, which contains a gene‐pool that is not present in the modern‐bred improved varieties that comprise subpopulation I1 (Higgins et al., 2021). The selected regions are listed in Table S7 and the functional annotation of each region is detailed in Table S8. These regions had a mean length of 584 kbp, covered 30 Mbp, which represents 8.13% of the rice genome, and contained 4576 genes (Table S9).

To cross‐validate these 52 regions selected in I5, we calculated the *F*_ST_ per SNP between the 43 samples in the I5 subpopulation and the 190 samples in the landrace subpopulations, I2, I3 and I4. The variation of *F*_ST_ and diversity along each chromosome are shown in Figure 3e,f. Both *F*_ST_ and diversity varied widely along the genome and did not show the clear peaks seen in the XP‐CLR score, but peaks can be seen in *F*_ST_ pattern coinciding with XP‐CLR peaks. This is clearest on chromosome 12 where *F*_ST_ and XP‐CLR score showed a similar pattern and the diversity scores showing the opposite pattern. The *F*_ST_ peaks (selection signatures) were in the range of ~0.6–0.9, while the average *F*_ST_ between subpopulations ranged between 0.14 and 0.23 (I1 vs. I2: 0.16, I1 vs. I3: 0.15, I1 vs. I4: 0.16, I1 vs. I5: 0.22, I2 vs I3: 0.18, I2 vs. I4: 0.16, I2 vs. I5: 0.23, I3 vs. I4: 0.17, I3 vs. I5: 0.23, I4 vs. I5: 0.21, I5 vs. I2/3/4: 0.18). Indica‐5 is the most differentiated one with average *F*_ST_ ranging between 0.18 and 0.23. Our aim was to localize regions in the genome with both high *F*_ST_ between the I5 subpopulation compared with the other Vietnamese landrace subpopulations and low diversity in the I5 subpopulation. High *F*_ST_ but low diversity would be expected in recently selected regions, as can be seen on chromosome 10. Chromosome 3 also showed this pattern and contained a large number of selected regions. The mean *F*_ST_ per gene for the 4576 genes selected in I5 is listed in Table S10, and the mean *F*_ST_ per selected region is shown in Table S7. The 1,983,066 heterozygous SNPs in subpopulations I2, I3, I4 and I5 had a mean *F*_ST_ of 0.185, and this mean value increased to 0.305 for the subset of 177,874 SNPs within the I5 selected regions.

We repeated the *F*_ST_ analysis using a SNP set generated against the Indica LIU XU (Accession IRGC 109232‐1) reference, a long‐read assembly that is a representative of the XI‐3B2 Indica subpopulation (Zhou et al., 2020). The results of this analysis are detailed in the Appendix S1. Briefly, we observed a very similar pattern and correlation between the *F*_ST_ results using either the LIU XU::IRGC 109232‐1 (XI‐3B2) or Nipponbare references (Correlation 0.954), both by comparing the mean *F*_ST_ per chromosome or along the 12 chromosome.

**FIGURE 4:**
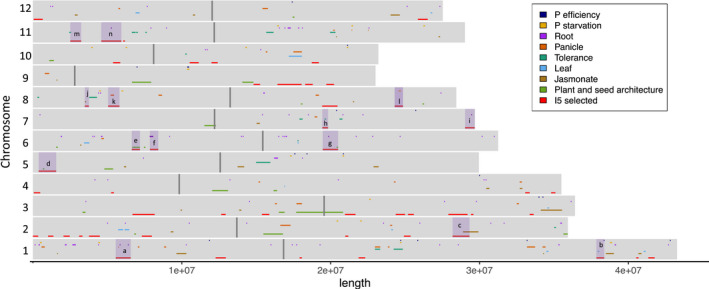
Vietnamese QTLs and their overlap with selected regions in the I5 subpopulation. QTLs from eight published studies (Higgins et al., 2021; Hoang, Gantet, et al., 2019; Hoang, van Dinh, et al., 2019; Mai et al., 2020; Phung et al., 2016; Ta et al., 2018; To et al., 2019, 2020) are plotted along each chromosome together with the 52 regions selected in the I5 subpopulation. The fourteen selected regions which overlap with at least one QTL are highlighted, the letters refer to the details shown in Table 2

The overlap of the 52 selected regions in the I5 subpopulation with the eight sets of QTLs is shown in Figure 4. Fourteen regions showed significant overlaps, these were shaded in Figure 4 and listed in Table 6, detailing the individual QTLs in Table S11. A comprehensive description of the overlaps for each region can be found in the Appendix S1. Candidate genes highlighted within these regions include the transcription factor *OsBLR1* (LOC_Os02g47660), which regulates leaf angle in rice via brassinosteroid signalling (Wang et al., 2020) in region ‘c’ and falls within the QTL for response of root length to jasmonate (qRTL1). Remarkably, *SSIIa* (LOC_Os06g12450) and *SDL/RNRS1* (LOC_Os06g14620) fall within regions ‘e’ and ‘f’, which overlap with two large regions selected during recent domestication by farmers in China. SSIIa is required for the edible quality of rice and plays an important role in grain starch synthesis (G. Zhang et al., 2011). *SDL/RNRS1* (LOC_Os06g14620) encodes the small subunit of ribonucleotide reductase, which is required for chlorophyll synthesis and plant growth development (Qin et al., 2017). The Auxin Response factor,*OsPILS2* (LOC_Os08g09190) falls within region ‘k’, which was selected in I3, I4 and I5, and coincides with two QTLs for panicle traits, primary branch number (PBN) and primary branch average length (PBL).

**TABLE 6:** Fourteen of the 52 regions under selection in the Indica I5 subpopulation, and their overlap with QTLs

Region	Chr.	Position (bp)	*F*_ST_ ^a^	Genes	Overlaps: Subpopulations^b^	Overlaps: Regions and genes^c^	Overlaps: QTLs^d^
I5_1	1	5,563,164–6,569,946	0.28	138	I2, I4, J1, J3, J4	1 (39)	Root mass (Phung et al., 2016) panicle morphology (Ta et al., 2018) (a)
I5_5	1	37,850,965–38,378,420	0.64	84	I1		Leaf mass (Hoang, Gantet, et al., 2019) Relative phosphate uptake efficiency (To et al., 2020) (b)
I5_16	2	28,191,142–29,329,745	0.24	168	I3		Jasmonate RTL (To et al., 2019) (c)
I5_30	5	386,347–1,563,159	0.28	190		3 (2)	9_PL (d)
I5_31	6	6,640,258–7,189,250	0.17	80	I1, I2, I4	1 (7), 3 (39)	12_GL (e)
I5_32	6	7,860,166–8,418,475	0.38	70	I3, I4, J3	1 (3), 3 (34)	Leaf length (Phung et al., 2016) (f)
I5_33	6	19,470,641–20,499,968	0.58	165	I1		Panicle length (Ta et al., 2018) root length and number (Phung et al., 2016) (g)
I5_34	7	19,443,608–19,825,988	0.19	54	I1, J4		Water content after drought (Hoang, van Dinh, et al., 2019) (h)
I5_35	7	29,030,233–29,677,525	0.76	97	I3		Root depth (Phung et al., 2016) (i)
I5_36	8	3,484,045–3,758,632	0.35	39	I3, I4		Jasmonate SHL (To et al., 2019) (j)
I5_37	8	5,052,017–5,809,093	0.38	127	I3, I4		Panicle branches (Ta et al., 2018) (k)
I5_39	8	24,300,313–24,859,863	0.23	92			Response of crown roots to phosphate (Mai et al., 2020) (l)
I5_48	11	2,510,079–3,239,747	0.38	109	I1, I4	1 (56)	Water content after drought (Hoang, van Dinh, et al., 2019) (m)
I5_49	11	4,590,276–5,937,318	0.35	200	J1	1 (3), 2 (14)	Root number (Phung et al., 2016) (n)

*Note*: Detailing the overlap of selected regions with published QTLs for Vietnamese rice populations, selected regions in Indica and Japonica subpopulations, and published selected regions (Cui et al., 2020; Lyu et al., 2014; Xie et al., 2015).

^a^ *F*_ST_ per region between the 43 samples in subpopulation I5 and the 190 samples in subpopulations I2, I3 and I4. Further details per region are available in Table S7.

^b^ Overlaps with regions selected in other subpopulations.

^c^ Number of genes in brackets. Numbers naming subpopulations from: 1, tall (Ind1) [Xie 2015]; 2, semi‐dwarf (IndII) [Xie 2015]; 3, Cui et al. (2020).

^d^ Letters naming QTLs plotted in Figure 4.

### 3.4 Candidate genes and nonsynonymous alleles in selected regions of I5

**FIGURE 5:**
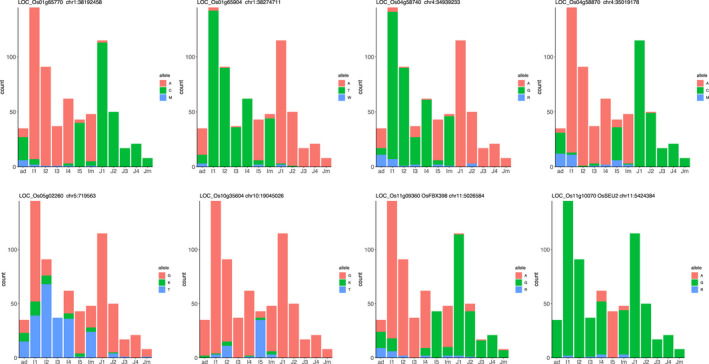
Allele Plots for “*High* impact” SNPs within eight candidate genes. Bar plots showing the base count for each subpopulation. A = adenine, T = thymine G = guanine, C = cytosine. Heterozygous calls are shown using IUPAC ambiguity codes

The final step was to complete a functional annotation of the 4576 genes in the 52 regions selected in the I5 subpopulation (Table S10) with the aim of identifying genes harboured within the selected regions relevant to breeding improvement. We were particularly interested in identifying genes which contain ‘*High* impact’ SNPs, which are SNPs predicted to cause deleterious gene effects, such as frame shifts, stop gains and start loses. The final list of 65 genes is detailed in Table 7, these were chosen based on the following three criteria (further details in Table S12); *F*_ST_ over 0.5 in the whole selected region or in the functionally enriched genes within regions, presence of ‘*High* impact’ SNPs, and the presence of candidate genes from overlapping QTL. Ten of the 65 genes contained ‘*High* impact’ SNPs. The alleles of eight of these genes were different in the I5 subpopulation compared with the other Indica subpopulations (Figure 5; Table S13). Among these eight genes, five of them showed the same allele as the Japonica subpopulations. However, two genes (LOC_Os10g35604 and LOC_Os11g10070/OsSEU2) had alleles unique to the I5 subpopulation.

**TABLE 7:** Functional annotation of the 65 candidate genes under selection in the Indica I5 subpopulation and overlap with genes selected in previous studies

Region	Gene ID (MSU)	*F*_ST_ ^a^	Gene name	Selected in^b^	SNP impact^c^	References	Gene function
I5_1	LOC_Os01g11860	0.300		2			DJ‐1 family protein, putative, expressed
I5_5	LOC_Os01g65670	0.909	OsAAP6|qPC1			Abbai et al. (2019), Peng et al. (2014)	Amino acid transporter, putative, expressed
I5_5	LOC_Os01g65770	0.936			Start lost		Expressed protein—rice specfic
I5_5	LOC_Os01g65904	0.788			Stop gained		Expressed protein—rice specfic
I5_5	LOC_Os01g66030	0.651	OsMADS2			Lombardo et al. (2017)	OsMADS2—MADS‐box family gene with MIKCc type‐box, expressed
I5_5	LOC_Os01g66070	0.445				To et al. (2019)	PHD‐finger domain containing protein, putative
I5_16	LOC_Os02g47310	0.564	VTE4			To et al. (2019)	Cyclopropane‐fatty‐acyl‐phospholipid synthase, putative, expressed
I5_16	LOC_Os02g47350	0.666				To et al. (2019)	Oxidoreductase, short‐chain dehydrogenase/reductase family, putative, expressed
I5_16	LOC_Os02g47400	0.501				To et al. (2019)	Pectinacetylesterase domain containing protein, expressed
I5_16	LOC_Os02g47410	0.522				To et al. (2019)	Protein kinase, putative, expressed
I5_16	LOC_Os02g47420	0.572	OSROPGEF			To et al. (2019)	ATROPGEF7/ROPGEF7, putative, expressed
I5_16	LOC_Os02g47440	0.536				To et al. (2019)	Syntaxin, putative, expressed
I5_16	LOC_Os02g47590	0.637				To et al. (2019)	Ornithine carbamoyltransferase, putative, expressed
I5_16	LOC_Os02g47660	0.372	OsBLR1			Wang et al. (2020)	Basic helix–loop–helix, putative, expressed
I5_17	LOC_Os03g12840	0.477	DSM3|OsITPK2		Stop gained	Du et al. (2011)	Inositol 1, 3, 4‐trisphosphate 5/6‐kinase, putative, expressed
I5_17	LOC_Os03g13010	0.837	TUD1|DSG1|ELF1			Sakamoto et al. (2017)	U‐box domain containing protein, expressed
I5_17	LOC_Os03g13140	0.879	Hb1			Lira‐Ruan et al. (2011)	Non‐symbiotic haemoglobin 2, putative, expressed
I5_17	LOC_Os03g14669	0.918	OsHAP5C			Kim et al. (2016)	Core histone H2A/H2B/H3/H4, putative, expressed
I5_23	LOC_Os03g49500	0.719	Os‐ERS1			Yu et al. (2017)	Ethylene receptor, putative, expressed
I5_23	LOC_Os03g51050	0.660	PTR8	1,3		Ouyang et al. (2010)	Peptide transporter PTR2, putative, expressed
I5_25	LOC_Os03g58600	0.844	MEL1			Yi et al. (2012)	PAZ domain containing protein, putative, expressed
I5_25	LOC_Os03g58630	0.886	OsTrxh4			Ying et al. (2017)	Thioredoxin, putative, expressed
I5_29	LOC_Os04g58740	0.818		2	Start lost		Expressed protein—rice specfic
I5_29	LOC_Os04g58750	0.815	OsBSK3	2		Zhang et al. (2016)	Protein kinase family protein, putative, expressed
I5_29	LOC_Os04g58780	0.806	WSL5|OsPPR4	2		Liu et al. (2018)	Pentatricopeptide repeat protein, putative, expressed
I5_29	LOC_Os04g58870	0.813			Splice acceptor or intron variant	Tu et al. (2015)	exo70 exocyst complex subunit, putative, expressed
I5_29	LOC_Os04g58880	0.826	RLS2|OsEXO70A1			Tu et al. (2015)	exo70 exocyst complex subunit, putative, expressed
I5_30	LOC_Os05g02260	0.617	bip130		Stop gained	Zhou et al. (2019)	Interacts with OsMPK1
I5_31	LOC_Os06g12450	0.360	ALK|SSIIa	4		Zhang et al. (2011)	Soluble starch synthase 2–3, chloroplast precursor, putative, expressed
I5_32	LOC_Os06g14620	0.471	*SDL/RNRS1*	4		Qin et al. (2017)	Ribonucleoside‐diphosphate reductase small chain, putative, expressed
I5_33	LOC_Os06g34360	0.959				Zang et al. (2016)	Zinc finger, C3HC4 type domain containing protein, expressed
I5_33	LOC_Os06g34650	0.948				Zang et al. (2016)	Zinc finger, C3HC4 type domain containing protein, expressed
I5_33	LOC_Os06g33520	0.509	OsABP			Macovei et al. (2012)	DEAD/DEAH box helicase, putative, expressed
I5_35	LOC_Os07g48560	0.927	WOX11			Zhang et al. (2018)	Homeobox domain containing protein, expressed
I5_35	LOC_Os07g48640	0.953	OsSDR			Kim et al. (2009)	Short‐chain dehydrogenase/reductase, putative, expressed
I5_35	LOC_Os07g48680	0.955				Zang et al. (2016)	Zinc finger, C3HC4 type domain containing protein, expressed
I5_35	LOC_Os07g48750	0.920	OsARAF1			Sumiyoshi et al. (2013)	Alpha‐N‐arabinofuranosidase, putative, expressed
I5_35	LOC_Os07g48780	0.907	OsCam1‐2|OsCam1			Saeng‐ngam et al. (2012), Yuenyong et al. (2018)	OsCam1‐2—Calmodulin, expressed
I5_35	LOC_Os07g48820	0.901	OsbZIP63|OsNIF1			Delteil et al. (2012), Vemanna et al. (2019)	Transcription factor, putative, expressed
I5_35	LOC_Os07g48830	0.931	OsGolS2|wsi76			Mukherjee et al. (2019)	Glycosyl transferase 8 domain containing protein, putative, expressed
I5_35	LOC_Os07g48920	0.916	OsALDH22			Yang et al. (2012)	Aldehyde dehydrogenase, putative, expressed
I5_36	LOC_Os08g06370	0.014				To et al. (2019)	MYB family transcription factor, putative, expressed
I5_37	LOC_Os08g09110	0.904			Stop gained		NB‐ARC domain containing protein, expressed
I5_37	LOC_Os08g09190	0.286	OsPILS2			Ta et al. (2018)	Auxin efflux carrier component, putative, expressed
I5_39	LOC_Os08g39100	0.239	OsPP2C66			Mai et al. (2020)	Protein phosphatase 2C, putative, expressed
I5_39	LOC_Os08g38990	0.202	OsWRKY30			Mai et al. (2020)	WRKY30, expressed
I5_41	LOC_Os09g28280	0.654		4			Gibberellin receptor GID1L2, putative, expressed
I5_41	LOC_Os09g28840	0.654					OsSCP43—Putative Serine Carboxypeptidase homologue, expressed
I5_42	LOC_Os09g30340	0.971	PSAG			Park et al. (2012)	Photosystem I reaction centre subunit, chloroplast precursor, putative, expressed
I5_42	LOC_Os09g30360	0.973					Caffeoyl‐CoA O‐methyltransferase, putative, expressed
I5_42	LOC_Os09g30380	0.966					AP005392‐AK108636—NBS/LRR genes that are S‐rich,divergent TIR, divergent NBS, expressed
I5_42	LOC_Os09g30400	0.954	OsWRKY80			Peng et al. (2016)	WRKY90, expressed
I5_42	LOC_Os09g30410	0.961					expressed protein
I5_42	LOC_Os09g31019	0.942				Chen et al. (2017)	Ubiquitin fusion protein, putative, expressed
I5_47	LOC_Os10g35260	0.703		3			Rf1, mitochondrial precursor, putative, expressed
I5_47	LOC_Os10g35540	0.783		3			Hydrolase, alpha/beta fold family domain containing protein, expressed
I5_47	LOC_Os10g35560	0.692	OsSFR6	3		de Freitas et al. (2019)	Expressed protein
I5_47	LOC_Os10g35604	0.661		3	Stop gained		Expressed protein
I5_47	LOC_Os10g35640	0.700	Rf1b	3			Rf1, mitochondrial precursor, putative, expressed
I5_48	LOC_Os11g05640	0.367	OsZIP‐2a|OsbZIP80	2		Nijhawan et al. (2008)	bZIP transcription factor domain containing protein, expressed
I5_48	LOC_Os11g06390	0.746	OsACTIN2	2			Actin, putative, expressed
I5_48	LOC_Os11g06410	0.841	SAB18	2			Homeodomain, putative, expressed
I5_48	LOC_Os11g06490	0.715					Ribosome inactivating protein, putative, expressed
I5_49	LOC_Os11g09360	0.919	OsFBX398		Splice acceptor or intron variant	Jain et al. (2007)	OsFBX398—F‐box domain containing protein, expressed
I5_49	LOC_Os11g10070	0.721	OsSEU2	3	Splice acceptor or intron variant	Tanaka et al. (2017)	Transcriptional corepressor SEUSS, putative, expressed

^a^ *F*_ST_ per region between the 43 samples in subpopulation I5 and the 190 samples in subpopulations I2, I3 and I4. Further details are available in Table S12.

^b^ 1, Ecotype differentiated genes (Lyu et al., 2014). 2, tall (Ind1) (Xie et al., 2015). 3, semi‐dwarf (IndII) (Xie et al., 2015). 4, domestication (Cui et al., 2020).

^c^ As measured by SNP effect.

## 4 DISCUSSION

Vietnam has one of the richest rice germplasm resources with over 4000 years of rice‐cultivating experience. Local farmers have bred varieties to suit their ecosystem and regional culinary preferences. These conscious and unconscious selection processes have resulted in detectable changes in allele frequencies at selected sites and their flanking regions. We used a well‐tested method, named XP‐CLR, to identify distorted allele frequency patterns in contiguous SNP sites that cannot be explained by random drift. To identify regions under selection, we leveraged the strong population structure recently described in Vietnam (Higgins et al., 2021), which comprised five Indica and four Japonica subpopulations of native rice accessions adapted to variable geography and latitude range.

We observed a stronger signature of selection in the Indica subtypes than in the Japonica subtypes, which may reflect the higher diversity within the Indica subtypes in Vietnam. Taking into consideration the size and diversity in each subpopulation (Table 1; Higgins et al., 2021), the whole‐genome XP‐CLR score was lower in the larger subpopulations (I1 and J1) and the subpopulations with the lower diversity. However, this trend was not true in the subpopulation indica‐5 (I5), which showed a higher selection score than the other subpopulations with comparable size and diversity. Within the Indica subtypes, the subpopulation I5 showed the highest XP‐CLR score against the subpopulation I1, which supports a strong signature for selection in I5 compared with the modern‐bred varieties in I1. On the contrary, the lowest XP‐CLR score was obtained when I5 was compared with the I3 subpopulation, which is adapted to upland ecosystems (Phung et al., 2014). This suggests I5 shares selection pressures and resilient traits with upland varieties. Intermediate XP‐CLR scores were obtained for the comparison of I5 with the two lowland subpopulations I2 (Mekong Delta) and I4 (Red River Delta).

Diversity is reduced when regions are under selection, but the observed diversity depends on many factors, including how long ago the selection occurred and the type of alleles selected alongside. This is referred to as the hitchhiking effect (Pavlidis & Alachiotis, 2017). The fixation index (*F*_ST_) is a measure of population differentiation due to genetic structure. Both measurements vary highly along the genome but can provide additional information about the selected regions identified using XP‐CLR. In this study, we calculated *F*_ST_ by comparing the I5 accessions to accessions in subpopulations I2, I3 and I4. We did not include the accessions in the elite I1 subpopulation, as we are specifically interested in genes that have been selected during the breeding of landraces within Vietnam. We used *F*_ST_ as a cross‐validation measure for identifying regions and genes under strong selection in the I5 subpopulation, and in support of the selection measurements obtained using XP‐CLR. While distinguishing the effect of selection (*F*_ST_ peaks) from population structure (averaged *F*_ST_) can be difficult in highly differentiated subpopulations, a comparison between averaged and local *F*_ST_ values evidenced this was not an issue in our study.

Assigning functional roles to both regions and genes within the regions was the following natural step to identify breeding targets. We used two approaches, overlap with QTLs and functional enrichment. Seven QTL studies have been carried out on this data set, finding associations for a range of traits relating to yield, this enables us to propose functional associations for around a third of the selected regions. A functional enrichment analysis evidenced selected regions were more clearly associated with specific GO terms in the Indica subpopulations than in the Japonica ones. The enrichment of GO terms was not correlated with the total number of genes or genome length in each subpopulation.

Looking in more detail at the 52 regions selected in the I5 subpopulation using a range of criteria, we identified 65 candidate genes within 20 of the selected regions. Six of these regions had a mean *F*_ST_ over 0.5 and we highlighted the following candidate genes within these regions. In region I5_35, we identified the transcription factor *WOX11* involved in crown root development (T. Zhang et al., 2018) and *OsCam1*, *OsbZIP63*, and *OsSDR*, which have putative roles in defence (Kim et al., 2009). Further genes of interest were (i) *OsAAP6*, a regulator of grain protein content (Peng et al., 2014), in region I5_5, (ii) *OsBSK3* (Zhang et al., 2016) and *WSL5* (Liu et al., 2018), which play roles in growth, in region I5_29, (iii) *OsABP,* which is upregulated in response to multiple abiotic stress treatments (Macovei et al., 2012), falls within region I5_33; and (iv) *OsSFR6*, a cold‐responsive gene (de Freitas et al., 2019), in region I5_47. In addition, eight of the ten genes containing ‘*high* impact’ mutations showed a different allelic content in the I5 subpopulation compared with the other Indica subpopulations, and in six cases these alleles were similar to the Japonica ones. Two genes containing ‘*high* impact’ mutations were *OsFBX398*, an F‐box gene with a potential role in both abiotic and biotic stresses (Jain et al., 2007; Vemanna et al., 2019), in region I5_49; and *bip130* (Zhou et al., 2019) in region I5_30, which regulates abscisic acid‐induced antioxidant defence and fall within our QTL for panicle length (9_PL). To pinpoint candidate genes for a range of agronomic traits, we looked for overlap of selected regions with relevant QTLs. 14 of the 52 regions selected in the I5 subpopulation had overlaps with a wide range of QTLs, two of the most relevant genes in these regions were *SSIIa*, which is responsible for the eating quality of rice (Zhang et al., 2011), and *OsbZIP80*, which is a transcription factor involved in dehydration stress response (Nijhawan et al., 2008).

Finally, we looked for overlaps with selected genes identified in three published studies using XP‐CLR in rice (Cui et al., 2020; Lyu et al., 2014; Xie et al., 2015). Lyu et al. (2014) identified 56 Indica‐specific genes in selected regions, which may account for the phenotypic and physiological differences between upland and irrigated rice. Thirty‐one of these genes were on chromosome 3 and lied within regions also selected in the I4 and I5 subpopulations (I5_23, I5_24). The gene with the highest *F*_ST_ (0.67) is *ptr8* (LOC_Os03g51050), which encodes a peptide transporter (Ouyang et al., 2010). Xie et al. (2015) identified 2125 and 2098 coding genes in regions selected in the Chinese landraces (IndI) and modern‐bred (IndII) subpopulations, respectively. We evidenced an overlap of 131 genes in selected regions in the I5 subpopulation with the genes selected in the IndI subpopulation and an overlap of 235 genes with the genes selected in the IndII subpopulation. This includes seven genes in I5_22 and two genes in I5_23, both regions on chromosome 3, which were selected in all three subpopulations. Cui et al. (2020) identified 186 potential selective‐sweep regions in the Indica subtypes, of which 33 overlap with nine of the 52 regions identified in the I5 subpopulation. These nine regions contained 153 genes (Table 2). Cui et al. were specifically addressing the role of indigenous farmers in shaping the population structure of rice landraces in China, there is the possibility that similar regions may also have been selected in Vietnam. Substantial overlaps were found in three regions. On chromosome 2, 3 regions overlapped with I5_14. On chromosome 6, 11 regions overlapped with I5_31 and I5_32, including gene SIIa (LOC_Os06g12450), which is an important agronomic gene which is responsible for the eating quality of rice and plays an important role in grain synthesis. On chromosome 9, 13 regions overlapped with I5_4, including gene LOC_Os09g28280, which is a putative gibberellin receptor GID1L2 detailed in Table 2.

XP‐CLR has proved a valuable method for identifying regions selected in the Vietnamese rice subpopulations and provided an insight into how natural selection and agricultural practices of farmers in Vietnam have shaped the population structure. Annotation of these regions with both overlaps with QTLs for a range of agronomic traits and functional enrichment allowed us to prioritize candidate regions as targets for breeding programs. Our results give further support for the Indica I5 subpopulation, which is essentially adapted to irrigated and rainfed lowland ecosystems, being an important source of novel alleles for both national and international breeding programmes. Using a range of criteria, *F*_ST_ and diversity in these regions, we identified 65 genes which could be further investigated for their breeding potential.

## CONFLICT OF INTEREST

The authors declare no conflicts of interest.

## Supporting information


Tables S1‐S13

Figure S1

Figure S2

Figure S3

Figure S4

Appendix S1

## Data Availability

All sequence data used in this manuscript have been deposited as study PRJEB36631 in the European Nucleotide Archive. Plant accessions were obtained from the Vietnamese National Genebank in compliance with the national laws and international treaties. A research collaboration was developed with scientists from the countries providing genetic samples, all collaborators are included as co‐authors, the results of the research have been shared with the provider stakeholders and the broader national and international scientific community.
