# Supplementary material for: Genomic regions and candidate genes selected during the breeding of rice in Vietnam
Source: Evol Appl. 2022 Jul 9;15(7):1141–61. doi: 10.1111/eva.13433 (PMC9309459; doi:10.1111/eva.13433)
Supplement: Supplementary file 2 — Figure S1 [file EVA-15-1141-s003.pdf]

Figure S1. Chromosome plots of regions selected in each Indica subpopulation showing the regions selected against each individual subpopulation and the shaded final selected regions which were selected against three subpopulations.

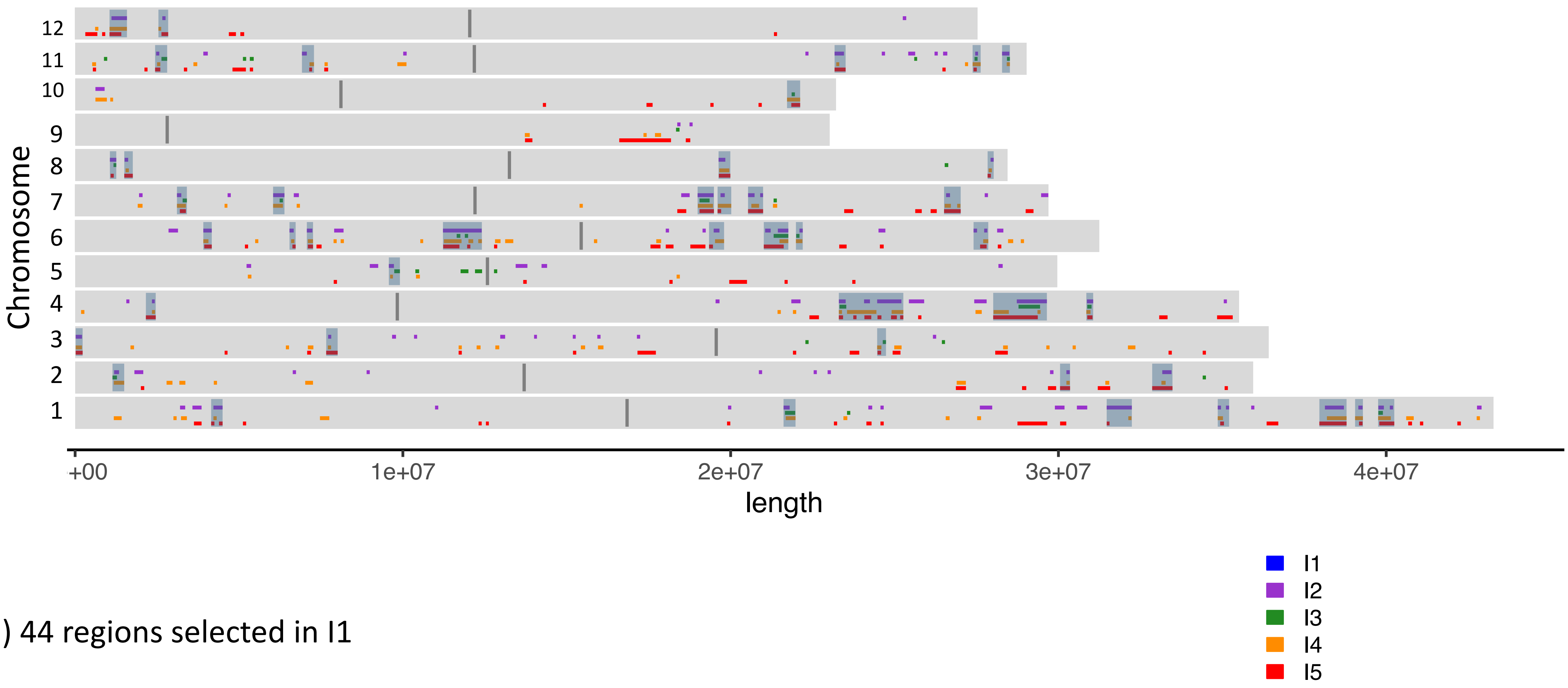

a) 44 regions selected in I1

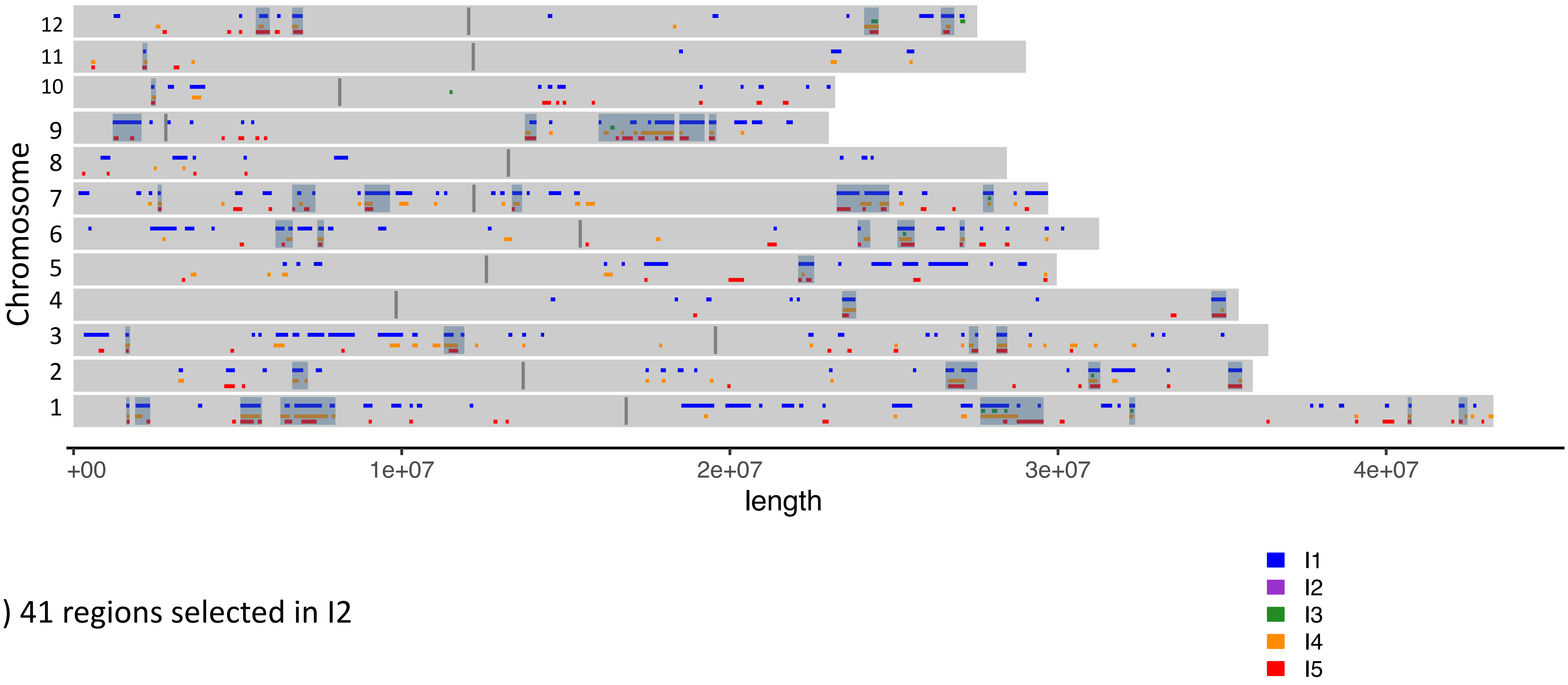

b) 41 regions selected in I2

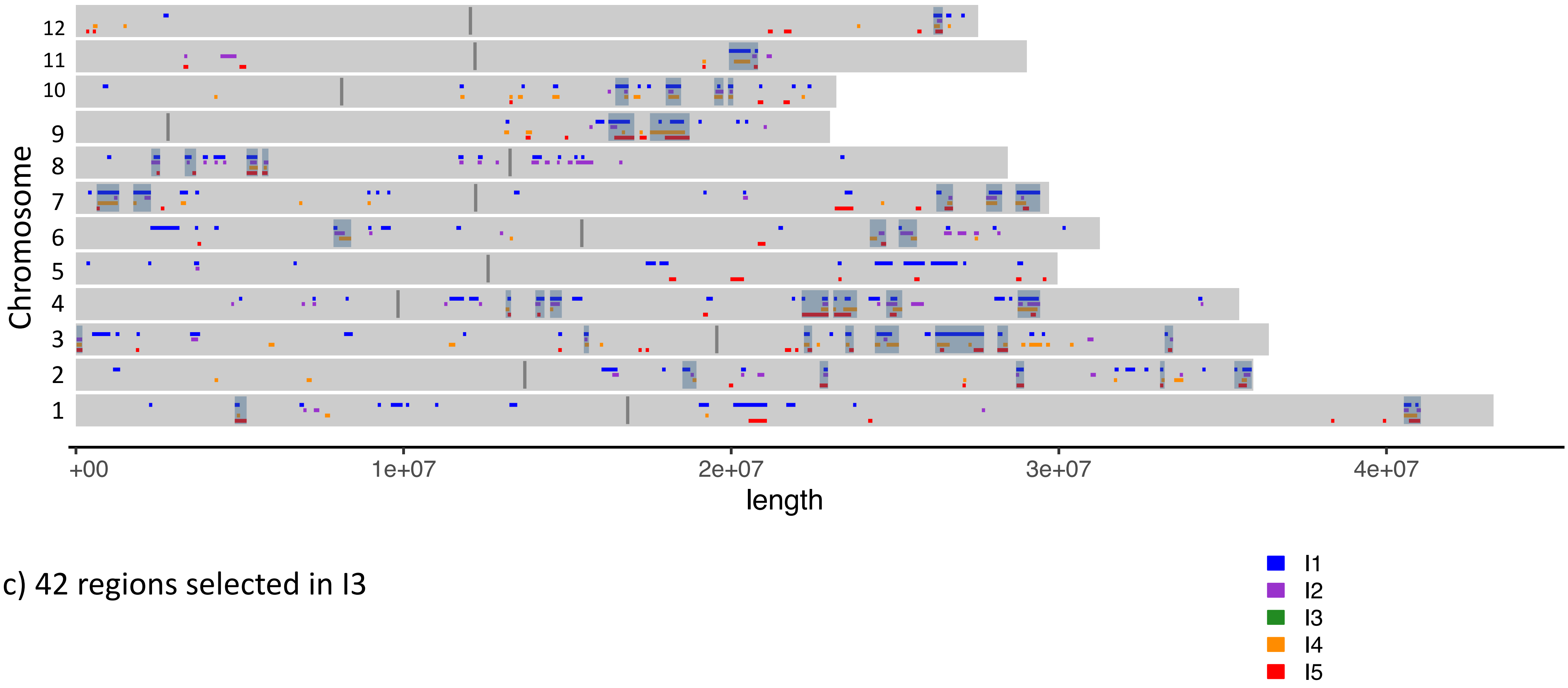

c) 42 regions selected in I3

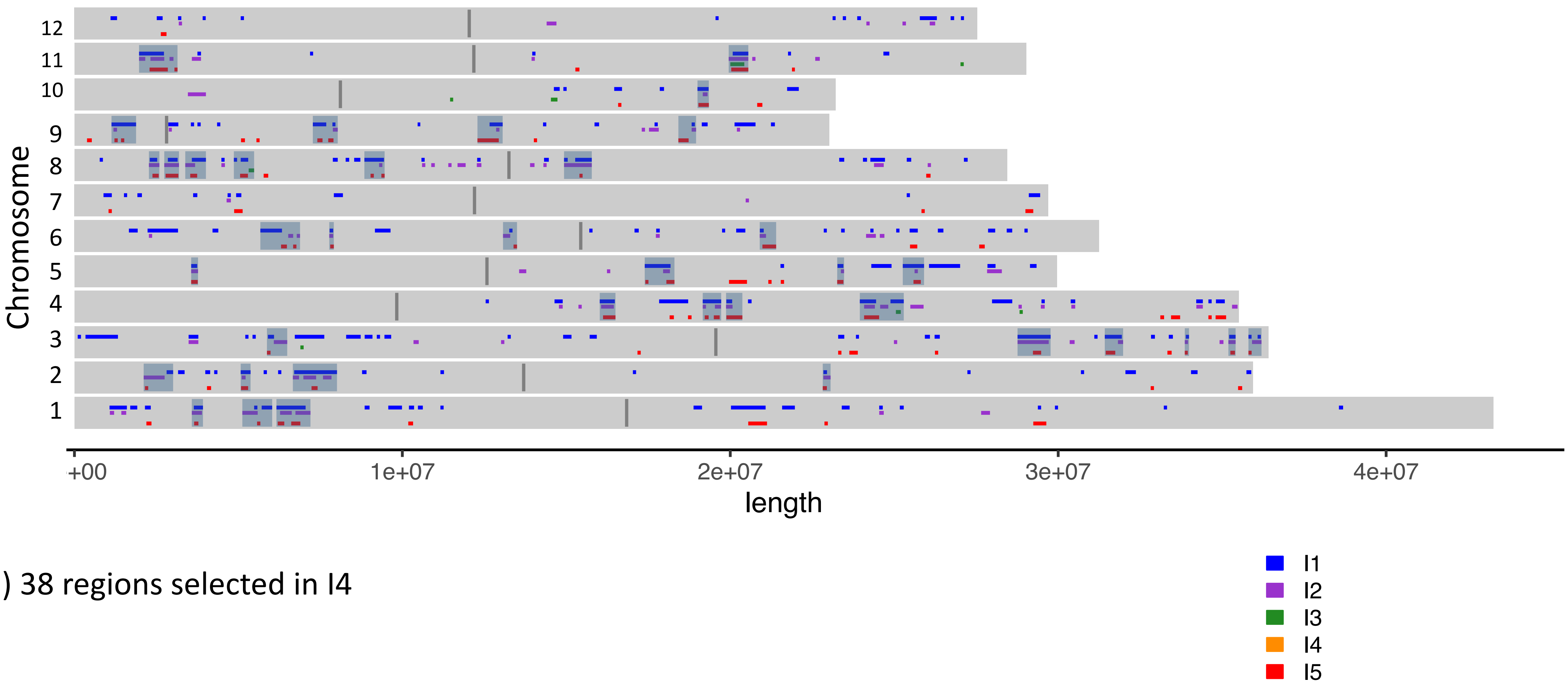

d) 38 regions selected in I4

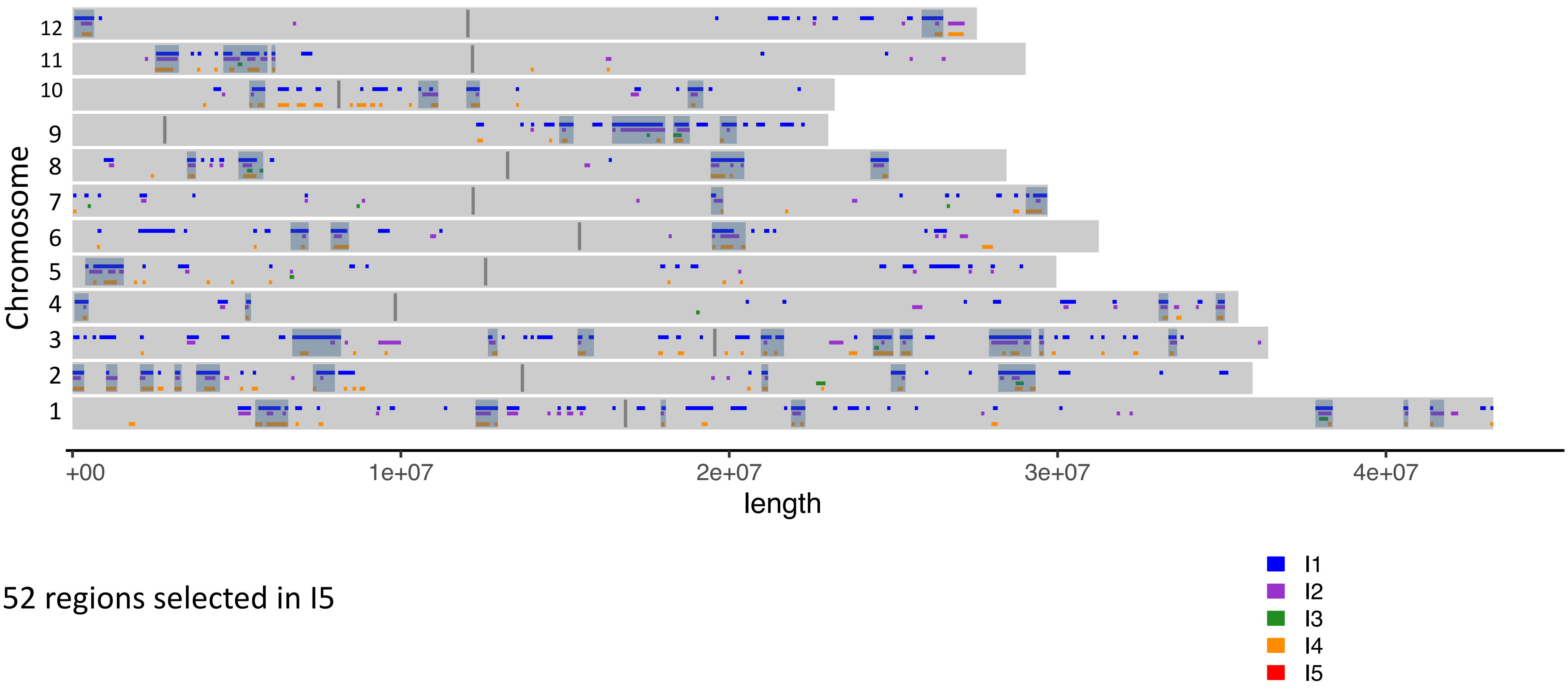

e) 52 regions selected in I5
