# Supplementary material for: Genomic regions and candidate genes selected during the breeding of rice in Vietnam
Source: Evol Appl. 2022 Jul 9;15(7):1141–61. doi: 10.1111/eva.13433 (PMC9309459; doi:10.1111/eva.13433)
Supplement: Supplementary file 4 — Figure S3 [file EVA-15-1141-s001.pdf]

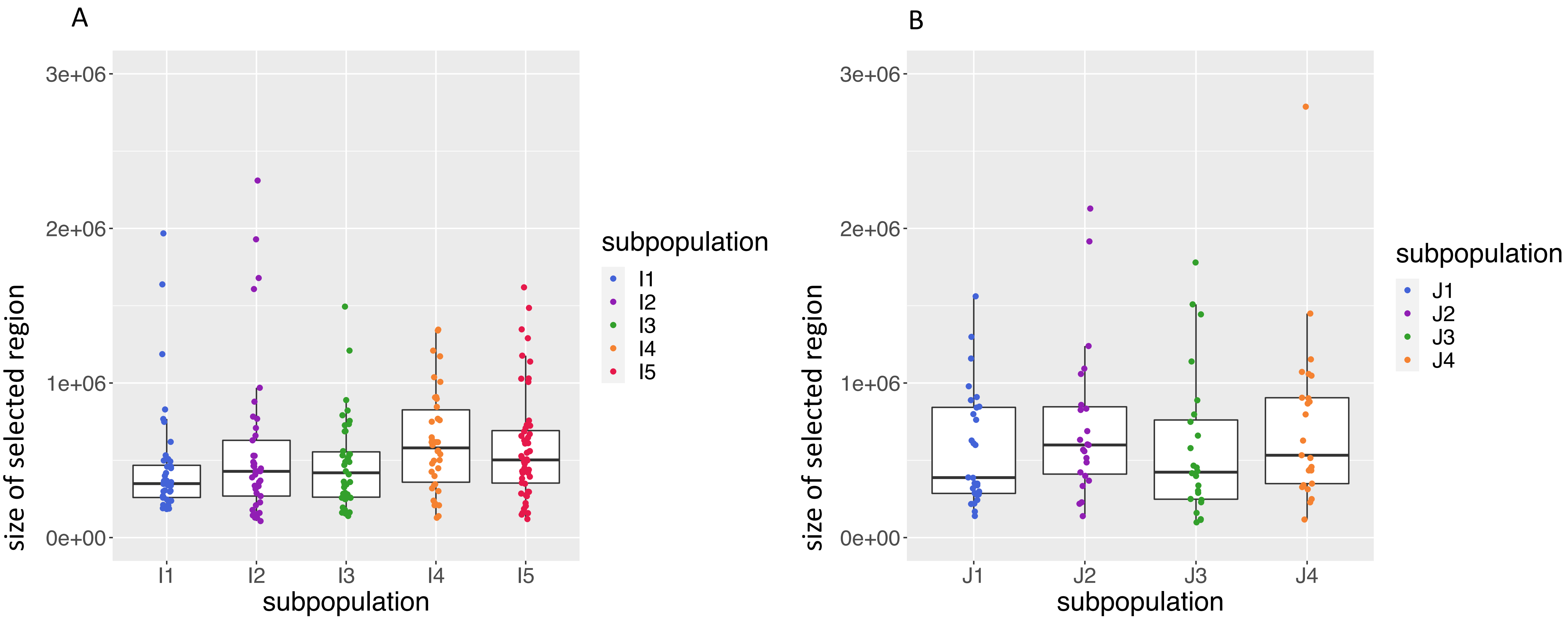

Fig. S3 Range of sizes for selected regions in (a) five Indica subpopulations and (b) four Japonica subpopulations
