# Supplementary material for: Genomic regions and candidate genes selected during the breeding of rice in Vietnam
Source: Evol Appl. 2022 Jul 9;15(7):1141–61. doi: 10.1111/eva.13433 (PMC9309459; doi:10.1111/eva.13433)
Supplement: Supplementary file 5 — Figure S4 [file EVA-15-1141-s002.pdf]

A

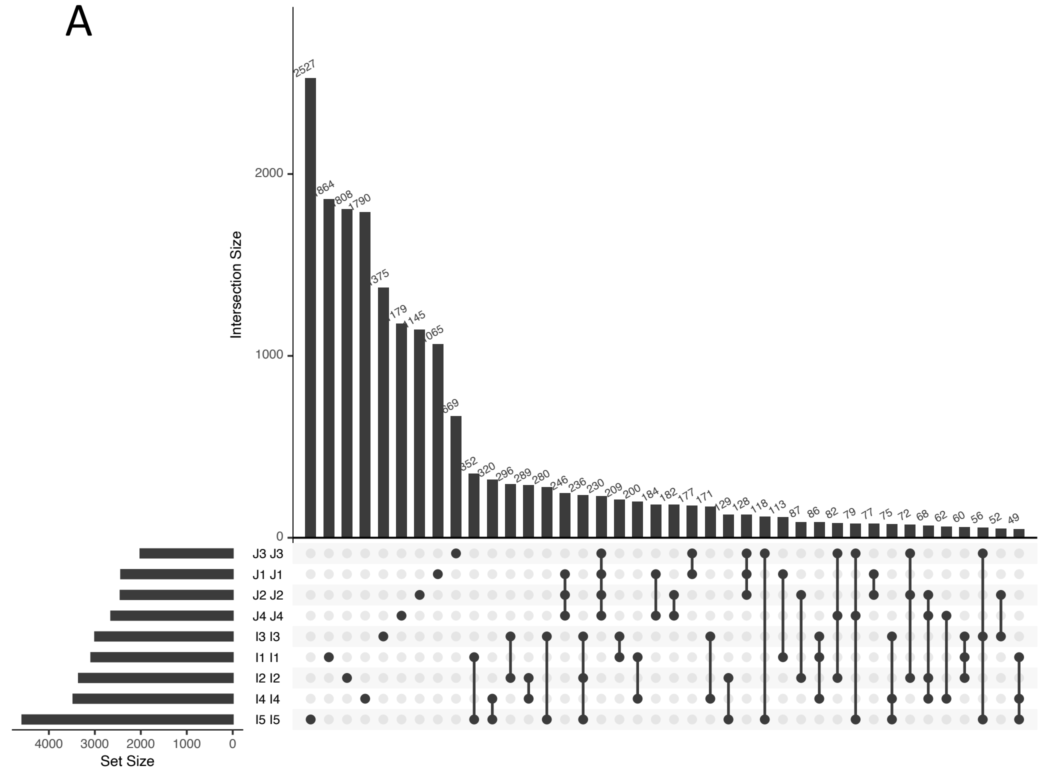

B

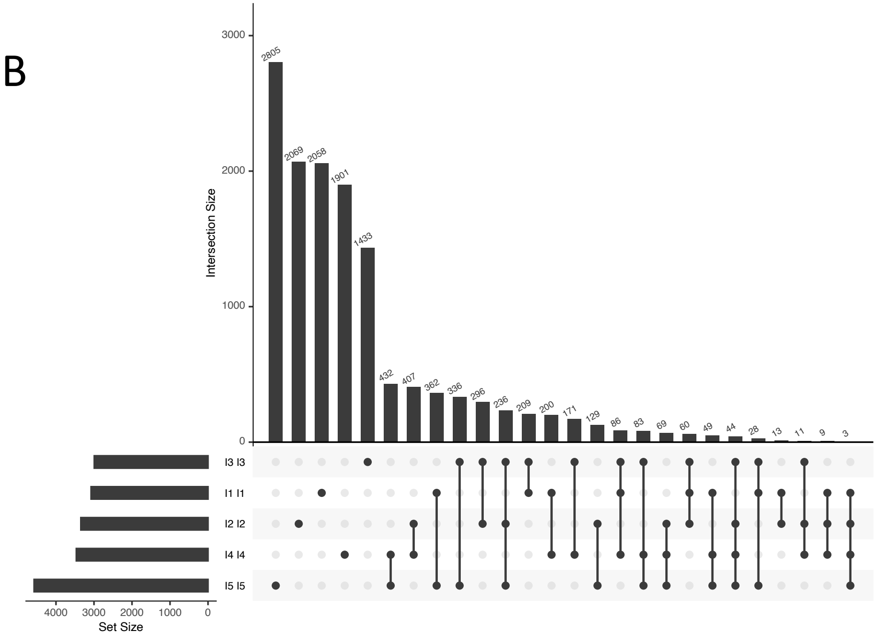

C

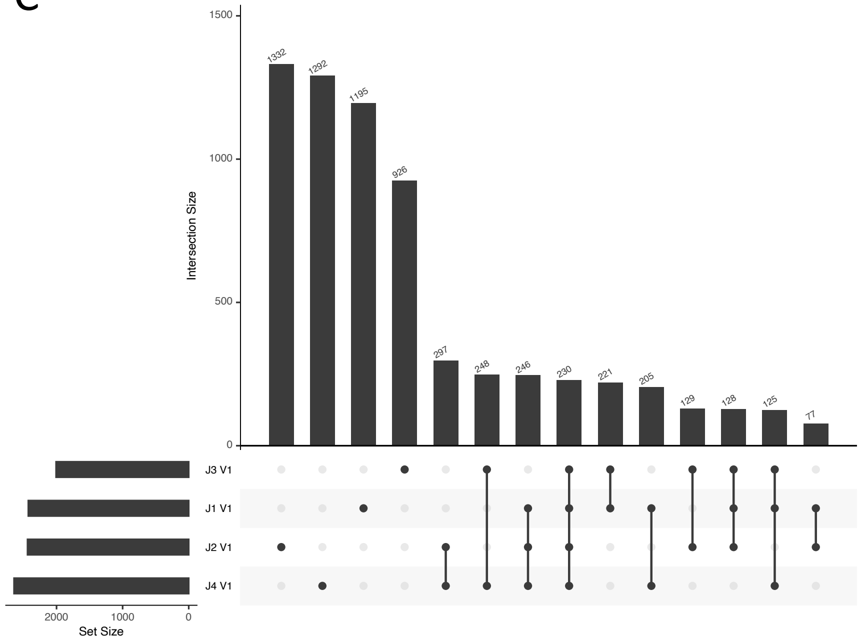

Fig. S4 Upset plots for overlap of genes in selected regions for (a) all nine subpopulations, (b) five Indica subpopulations and (c) four Japonica subpopulations
