# Supplementary material for: Genomic regions and candidate genes selected during the breeding of rice in Vietnam
Source: Evol Appl. 2022 Jul 9;15(7):1141–61. doi: 10.1111/eva.13433 (PMC9309459; doi:10.1111/eva.13433)
Supplement: Supplementary file 6 — Appendix S1 [file EVA-15-1141-s005.docx]

**Supplementary Appendix**

1. Verification of F_ST_ results obtained using the Japonica Nipponbare reference for the Indica dataset using a SNP set aligned to the Indica LIU XU::IRGC 109232-1 reference.
2. The proportion of each subpopulation that originated from each of the eight administrative regions of Vietnam.
3. Additional information on the selected regions in the outlying Indica-5 (I5) subpopulation

**1/ Verification of F_ST_ results obtained using the Japonica Nipponbare reference for the Indica dataset using an SNP set aligned to the Indica LIU XU::IRGC 109232-1 reference.**

**Method**

LIU XU::IRGC 109232-1 was selected as the Indica reference as it belongs to the XI-3B2 Indica subpopulation (Zhou et al. 2020), which is well represented in Vietnam. The 379 Indica samples (Table 1) from the 612 Vietnamese dataset described in Higgins et al. (2021) were aligned to the LIU XU::IRGC 109232-1 Indica reference (BioProject: PRJNA577228, BioSample: SAMN13021815) with BWA-MEM v0.7.17 with the flag -M, duplicate reads were later annotated with Picard tools v2.1.1. The Bam files were sorted and merged using SAMtools v1.7. Variant calling was carried out using the merged bam file for the 379 samples using FreeBayes v1.0.2 with --min-coverage 10. These SNPs were filtered using VCFtools v0.1.13 for bi-allelic SNPs, Q30, mac 3, then further filtered for MAF 5% and sites missing in up to 20% of the calls.

Principal component analysis was done using Tassel v5.2.41. The Japonica impute SNP set (Higgins et al. 2021) and the Indica SNP set were further filtered to keep the 203 samples in subpopulations I2, I3, I4 and I5. F_ST_ analysis was carried out using the function *snpgdsFst*  from the R package SNPRelate v1.24.0. F_ST_ per SNP was averaged over 100,000bp sliding windows using BEDtools v2.26.0.

The “Indica LIU XU SNP set” refers to the set of SNPs with up to 20% missing data obtained by alignment to the LIU XU::IRGC 109232-1 reference. The “Indica Nipponbare SNP set” refers to the set of imputed SNPs described in Higgins et al. (2021) and obtained by alignment to the Japonica Nipponbare (IRGSP-1.0) reference.

**Results**

The average mapping rate (% alignment for properly paired reads) per subpopulation was 93.6%, 93.4%, 93.2%, 94.5% and 93.9% for I1, I2, I3, I4 and I5, respectively. Comparing the mapping rate between the Japonica and Indica references, the Indica samples showed an increase in mapping rate from an average of 90.26% for the Japonica reference to 93.70% for the Indica reference. The increase in mapping rate ranged from 1.26% to 5.13%, the average per subpopulation was smallest for the I5 subpopulation (3.06%) and largest for the I4 subpopulation (3.95%). As expected, the highest mapping rate was obtained for the I4 subpopulation, which was shown in Higgins et al. (2021) to fall within the XI-3B2 subpopulation.

14,076,858 variants were obtained for the 379 samples; these were filtered to obtain 4,623,880 bi-allelic SNPs with a minimum allele count of 3 and a quality value above 30. This SNP set was further filtered for missing in a maximum of 20% of samples and a minor allele frequency of 5% to obtain a set of 2,073,909 SNPs. Principal component analysis (Figure) confirmed that the population structure was similar to that obtained in Higgins et al. (2021) for 426 samples using the Japonica reference.


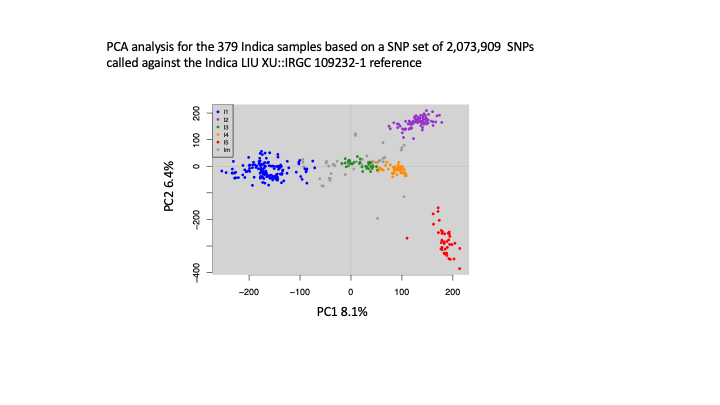


F_ST_ between the I5 and the I2, I3, I4 subpopulations was used as a primary method of selecting genes of interest, which resulted in 4,576 genes selected in 52 regions in the I5 subpopulation (Results section in the main text). The correlation between mean F_ST_ per chromosome for the Indica LIU XU SNP set and the Indica Nipponbare SNP set was 0.954. F_ST_ results generally correlate with the length of selected regions obtained using XP-CLR for the 12 chromosomes; the correlation was 0.703 for the Indica Nipponbare SNP set and 0.696 for the Indica LIU XU SNP set. The following figure shows the mean F_ST_ per 100,000kb windows in steps of 10,000bp along the 12 chromosomes between the Indica Nipponbare and Indica LIU XU SNP sets


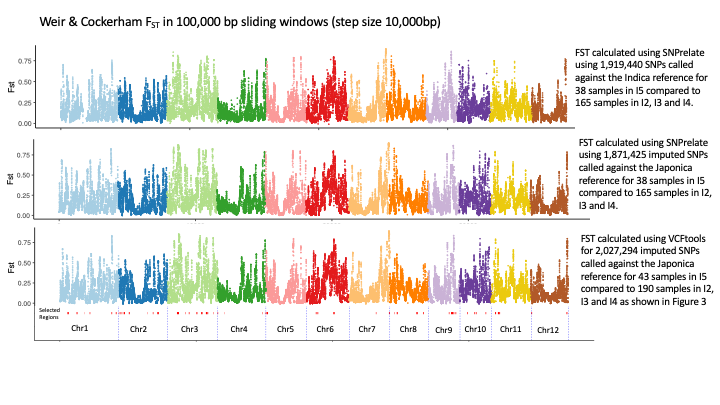


F_ST_ patterns are very similar between the three datasets except on chromosome 10, where the pattern is reversed in the Indica LIU XU SNP set. This is a consequence of chromosome 10 being inverted between the Indica and Japonica reference (Figure). This does not affect the list of selected loci.


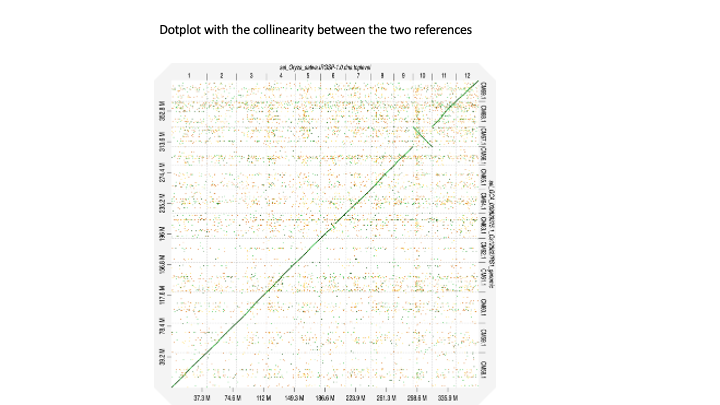


**2/ The proportion of each subpopulation which originated from each of the eight administrative regions of Vietnam.**

Data comprises a subset of 377 samples, 54% of Indica samples and 85% of Japonica samples.

The Ecosystem characteristics of the regions are as follows (Phung et al. 2014):

- The Mekong Delta and Red River Delta are a mixture of irrigated and rainfed lowlands.
- The North Central Coast, Northeast and South Central Coast are a mixture of upland and rainfed lowland.
- The Southeast is a mixture of upland and irrigated.
- The Northwest is mainly upland.


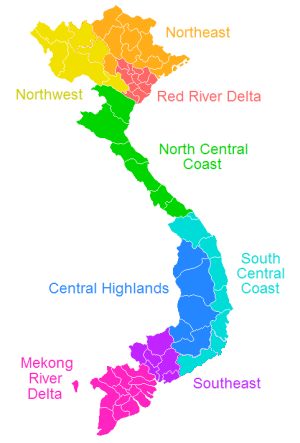


Japonica

Indica

**3/ Additional information on the selected regions in the outlying Indica-5 (I5) subpopulation.**

Overall, the I5 subpopulation had the highest XP-CLR selection scores; this is reflected in I5 having the greatest number of selected regions covering the highest proportion of the genome. I5 is an outlier subpopulation, which contains a gene pool not present in the modern bred improved varieties that comprise subpopulation I1 (Higgins et al., 2021). The XP-CLR score of the I5 subpopulation compared to the other four Indica subpopulations in 100 kbps windows is shown in Fig. 3. A cut-off XP-CLR score of 440 was used to define selected regions in I5; I5 vs I1 produced 207 regions with a mean length of 267 kbp (14.8% of the genome); I5 vs I2 produced 120 regions with a mean length of 204 kbp (6.57% of the genome); I5 vs I3 produced 14 regions with a mean length of 162 kbp (0.61% of the genome); I5 vs I4 produced 122 regions with a mean length of 122 kbp (6.02% of the genome). Regions selected in three or more subpopulations were merged to give 52 selected regions in I5. The regions are listed in Supplementary Table S7, and the functional annotation of each region is detailed in Supplementary Table S8. These regions had a mean length of 584 kbp, covered 30 Mbp, representing 8.13% of the rice genome, and contained 4,576 genes (Supplementary Table S9).

To gain further information on the uniqueness of these 52 regions selected in I5, we calculated the F_ST_ per SNP between the 43 samples in the I5 subpopulation and the 190 samples in the landrace subpopulations I2, I3 and I4. The variation of F_ST_ and diversity along each chromosome are shown in Fig. 3e and 3f. Both F_ST_ and diversity varied widely along the genome and did not show the clear peaks seen in the XP-CLR score, but peaks can be seen in the F_ST_ pattern coinciding with XP-CLR peaks. This is clearest on chromosome 12, where F_ST_ and XP-CLR scores showed a similar pattern, and the diversity scores showed the opposite pattern. We aimed to localise regions in the genome with both high F_ST_ between the I5 subpopulation and low diversity in the I5 subpopulation compared to the other Vietnamese subpopulations. High F_ST_ but low diversity would be expected in recently selected regions, as shown on chromosome 10. Chromosome 3 also showed this pattern and contained many selected regions. The mean F_ST_ per gene for the 4,576 genes selected in I5 is listed in Supplementary Table S10, and the mean F_ST_ per selected region is shown in Supplementary Table S7. The 1,983,066 heterozygous SNPs in subpopulations I2, I3, I4 and I5 had a mean F_ST_ of 0.185, and this mean value increased to 0.305 for the subset of 177,874 SNPs within the I5 selected regions.

The overlap of the 52 selected regions in the I5 subpopulation with the eight sets of QTLs is shown in Fig. 4. Fourteen regions showed significant overlaps; these were shaded in Fig. 4 and listed in Table 4, detailing the individual QTLs in Supplementary Table S11. Region ‘a’ was selected in I2, I4, I5, J1, J3 and J4, and coincided with a QTL for spikelet number (SpN) and two QTLs for root traits (q73, q84) where the following candidate genes LOC_Os01g10900, LOC_Os01g11010 and LOC_Os01g11860 were located. Region ‘b’ was selected in I1 and I5 and coincided with a QTL for Leaf weight (FW_TW) and a QTL for relative phosphate uptake (qRPUpE1.5) where the candidate gene LOC_Os01g66070 was located. Region ‘c’ was selected in I3 and I5 and overlapped a QTL for the response of root length to jasmonate (qRTL1), an overlap of 37 candidate genes, including the transcription factor *OsBLR1* (LOC_Os02g47660), which regulates leaf angle in rice via brassinosteroid signalling (Wang et al. 2020). Region ‘d’ partially overlapped a QTL for panicle length (9_PL) and a region selected during recent domestication by farmers in China (Cui et al. 2019). Region ‘e’ entirely overlapped a QTL for grain length (12_GL). Region ‘f’ was selected in I3, I4 and I5 and coincided with a QTL for leaf length (Rq2), which is only found in the Japonica subtype. Both regions ‘e’ and ‘f’ overlapped with two large regions selected during recent domestication by farmers in China. Gene *SSIIa* (LOC_Os06g12450) and *SDL/RNRS1* (LOC_Os06g14620) fall within this region. SSIIa is required for the edible quality of rice and plays a vital role in the grain starch synthesis (Zhang et al. 2011). *SDL/RNRS1* (LOC_Os06g14620) encodes the small subunit of ribonucleotide reductase, which is required for chlorophyll synthesis and plant growth development (Qin et al. 2017). Region ‘g’ was selected in I1 and I5 and coincided with a QTL for panicle length (14_PL) and a QTL for maximum root length (Rq35). Region ‘h’ was selected in J4, I1 and I5 and coincided with a QTL for relative water content (Tq7) observed after three weeks of drought stress. Region ‘i’ was selected in I3 and I5 and coincided with a QTL for root depth (Rq25). Region ‘j’ was selected in I3, I4 and I5, and overlapped with a QTL for the response of shoot length to jasmonate (qSHL4) and four candidate genes, including a G2-like transcription factor (LOC_Os08g06370). Region ‘k’ was selected in I3, I4 and I5, and coincided with two QTLs for panicle traits, primary branch number (PBN) and primary branch average length (PBL) that include the gene Auxin Response factor, *OsPILS2* (LOC_Os08g09190). Region ‘l’ coincided with a QTL for the number of crown roots in response to Pi deficiency (qNCR8.13), which contained two candidate genes, *OsPP2C66* (LOC_Os08g39100) encoding PHOSPHATASE 2C and transcription factor *OsWKKY30* (LOC_Os08g38990). Region ‘m’ was selected in I1, I4 and I5 and coincided with four QTLs related to the response of plants to drought (Tq12), which contained *OsbZIP80* (LOC_Os11g05640), a transcription factor involved in the dehydration stress response (Nijhawan et al. 2008). Region ‘n’ was selected in J1 and I5 and coincided with a QTL for a number of crown roots (Rq43).

Browning, B. L., and S. R. Browning. 2016. 'Genotype Imputation with Millions of Reference Samples', *Am J Hum Genet*, 98: 116-26.

Cui, Di, Hongfeng Lu, Cuifeng Tang, Jinmei Li, Xinxiang A, Tengqiong Yu, Xiaoding Ma, Enlai Zhang, Yanjie Wang, Guilan Cao, Furong Xu, Yongli Qiao, Luyuan Dai, Ruiqiang Li, Shilin Tian, Hee‐Jong Koh, and Longzhi Han. 2019. 'Genomic analyses reveal selection footprints in rice landraces grown under on‐farm conservation conditions during a short‐term period of domestication', *Evolutionary Applications*, 13: 290-302.

Garrison, E, ; Marth, G. 2012. 'Haplotype-based variant detection from short-read sequencing'.

Mansueto, L., R. R. Fuentes, F. N. Borja, J. Detras, J. M. Abriol-Santos, D. Chebotarov, M. Sanciangco, K. Palis, D. Copetti, A. Poliakov, I. Dubchak, V. Solovyev, R. A. Wing, R. S. Hamilton, R. Mauleon, K. L. McNally, and N. Alexandrov. 2017. 'Rice SNP-seek database update: new SNPs, indels, and queries', *Nucleic Acids Res*, 45: D1075-D81.

Mansueto, Locedie, Roven Rommel Fuentes, Dmytro Chebotarov, Frances Nikki Borja, Jeffrey Detras, Juan Miguel Abriol-Santos, Kevin Palis, Alexandre Poliakov, Inna Dubchak, Victor Solovyev, Ruaraidh Sackville Hamilton, Kenneth L. McNally, Nickolai Alexandrov, and Ramil Mauleon. 2016. 'SNP-Seek II: A resource for allele mining and analysis of big genomic data in Oryza sativa', *Current Plant Biology*, 7-8: 16-25.

Nijhawan, A., M. Jain, A. K. Tyagi, and J. P. Khurana. 2008. 'Genomic survey and gene expression analysis of the basic leucine zipper transcription factor family in rice', *Plant Physiol*, 146: 333-50.

Phung, N. T., C. D. Mai, P. Mournet, J. Frouin, G. Droc, N. K. Ta, S. Jouannic, L. T. Le, V. N. Do, P. Gantet, and B. Courtois. 2014. 'Characterization of a panel of Vietnamese rice varieties using DArT and SNP markers for association mapping purposes', *BMC Plant Biol*, 14: 371.

Qin, R., D. Zeng, R. Liang, C. Yang, D. Akhter, M. Alamin, X. Jin, and C. Shi. 2017. 'Rice gene SDL/RNRS1, encoding the small subunit of ribonucleotide reductase, is required for chlorophyll synthesis and plant growth development', *Gene*, 627: 351-62.

Wang, K., M. Q. Li, Y. P. Chang, B. Zhang, Q. Z. Zhao, and W. L. Zhao. 2020. 'The basic helix-loop-helix transcription factor OsBLR1 regulates leaf angle in rice via brassinosteroid signalling', *Plant Mol Biol*, 102: 589-602.

Zhang, G., Z. Cheng, X. Zhang, X. Guo, N. Su, L. Jiang, L. Mao, and J. Wan. 2011. 'Double repression of soluble starch synthase genes SSIIa and SSIIIa in rice (Oryza sativa L.) uncovers interactive effects on the physicochemical properties of starch', *Genome*, 54: 448-59.
